# Modeling the dynamics and kinetics of HIV-1 Gag during viral assembly

**DOI:** 10.1371/journal.pone.0196133

**Published:** 2018-04-20

**Authors:** Michael D. Tomasini, Daniel S. Johnson, Joshua S. Mincer, Sanford M. Simon

**Affiliations:** 1 Laboratory of Cellular Biophysics, Rockefeller University, New York, New York, United States of America; 2 Department of Physics and Astronomy, Hofstra University, 151 Hofstra University, Hempstead, New York, United States of America; 3 Department of Anesthesiology, Icahn School of Medicine at Mount Sinai, New York, New York, United States of America; University of Alabama at Birmingham, UNITED STATES

## Abstract

We report a computational model for the assembly of HIV-1 Gag into immature viral particles at the plasma membrane. To reproduce experimental structural and kinetic properties of assembly, a process occurring on the order of minutes, a coarse-grained representation consisting of a single particle per Gag molecule is developed. The model uses information relating the functional interfaces implicated in Gag assembly, results from cryo electron-tomography, and biophysical measurements from fluorescence microscopy, such as the dynamics of Gag assembly at single virions. These experimental constraints eliminated many classes of potential interactions, and narrowed the model to a single interaction scheme with two non-equivalent interfaces acting to form Gags into a hexamer, and a third interface acting to link hexamers together. This model was able to form into a hexameric structure with correct lattice spacing and reproduced biologically relevant growth rates. We explored the effect of genomic RNA seeding punctum growth, finding that RNA may be a factor in locally concentrating Gags to initiate assembly. The simulation results infer that completion of assembly cannot be governed simply by Gag binding kinetics. However the addition of membrane curvature suggests that budding of the virion from the plasma membrane could factor into slowing incorporation of Gag at an assembly site resulting in virions of the same size and number of Gag molecules independent of Gag concentration or the time taken to complete assembly. To corroborate the results of our simulation model, we developed an analytic model for Gag assembly finding good agreement with the simulation results.

## Introduction

A critical step in the life cycle of type 1 human immunodeficiency virus (HIV-1) is the assembly at the plasma membrane of a membrane associated viral particle containing the viral proteins and nucleic acids necessary to form an infectious virus. The main structural protein needed to assemble the viral particles is the polyprotein Gag, which is composed of four folded domains and two spacer regions. The matrix (MA) domain is myristoylated at its N-terminus and also contains a series of basic amino acids for targeting to and binding of Gag at the plasma membrane. The capsid (CA) domain is thought to be a region of Gag that significantly contributes to multimerization and can be further divided into the CA N-terminal domain (CA-NTD) and the CA C-terminal domain (CA-CTD) which are separated by a small, flexible linker. Flanking the capsid domain is the spacer domain SP1 followed by the nucleocapsid (NC) domain which contains two zinc-finger motifs involved in binding RNA. After NC is the spacer domain SP2 and the C-terminal P6 domain responsible for associating with the ESCRT machinery, which act in scission of the viral bud from the plasma membrane [1].

Following translation, Gag accumulates in the cytoplasm up to a concentration of ~500 nM, after which Gag aggregates can be detected stably associated with the plasma membrane [2]. Gag reaches the membrane through two pathways: direct recruitment to the membrane as single monomers or low-order multimers [3] or together with viral genomic RNA [4]. The genome is observed to be recruited to the plasma membrane prior to Gag assembly [5], possibly acting as a seed for an assembly site, although once an assembly site is observed, the time course for assembly is independent of the presence of HIV genome [5]. Once at the plasma membrane, Gag becomes anchored by insertion of its N-terminal myristoylation segment into the membrane and interaction of the highly basic region of MA with the acidic phospholipid phosphatidylinositol-(4,5)-bisphosphate (PIP_2_) contained in lipid raft domains [6]. The concentration of Gag at assembly sites increases over a period of 5–25 minutes [7,8] driven by putative interactions mediated through the CA domain. The assembling Gag forms into a continuous hexameric lattice while the spherical viral particle buds outward from the surface of the plasma membrane. At the end of the recruitment of Gag, the ESCRT machinery is recruited to the site of assembly [9,10,11] and scission of the viral particle takes place. HIV-1 Gag mutants which are defective in the P6 domain, and therefore do not recruit the ESCRT machinery, still fully assemble but do not scission off from the membrane. A fully formed immature virus particle has a diameter in the range of 120–150 nm [13]. It is estimated that with a complete Gag shell surrounding its inner surface, a fully formed immature virus particle would contain approximately 5000 Gag molecules [14]. However, it has been shown that virus particles do not always form a complete Gag shell, possibly as a result of the ESCRT machinery scissioning the viral bud prior to full assembly, resulting in estimates between 2400 to 5000 Gags per virion [12,14,15]. The HIV-1 protease cleaves Gag at five locations, presumably following scission of the viral particle from the plasma membrane. This results in the formation of the mature conical capsid consisting of approximately 1500 CA molecules assembled around the NC associated viral genome to make an infectious viral particle [13].

Experimental observations of HIV-1 assembly using total internal reflection fluorescence (TIRF) microscopy [7] set a number of constraints on any simulations of Gag assembly. First, puncta of Gag assemble at the plasma membrane on the timescale of 5–25 minutes. Second, as infection proceeded the time for viral assembly decreased, implying that higher concentrations of HIV-1 Gag at the plasma membrane result in faster viral particle assembly [7]. Third, at the temporal midpoint of the assembly, the growth rate was linear [7]. Fourth, assuming as an upper bound that the fully assembled virion has 5000 Gags at completion and a lower bound of 2500 Gags, if the growth rate is linear the growth rate is between 5 and 10 Gags per second. The fluorescence intensity of each punctum formed was similar indicating that assembled virus particles contain roughly the same number of Gag molecules.

The fluorescence intensity was not sufficient to visualize individual Gag molecules, and thus it is unclear the mechanism by which Gag molecules are added to an assembling virus particle. It is possible that Gags are first targeted to the plasma membrane as monomers and then laterally diffuse to the assembly site. Gags could form small aggregates at the plasma membrane which then combine or are added to a growing punctum. Alternatively, Gags could be added directly from the cytoplasm to an assembly site [8].

Recent investigations into the structure of the immature virus particle used cryoelectron tomography (cryo-ET) with subtomogram averaging and fitting of crystal structures of individual Gag domains into the cryo-ET densities [16]. Results show that Gag in immature particles forms a lattice of hexamers with a void space in the center of the hexamer and a spacing of ~8 nm between hexamer centers. In the cryo-ET reconstructions, no order was observed for MA or NC, but it has been proposed that MA alone assembles into ordered structures [17]. The main stabilizing interactions from the cryo-ET data were present in the CA and SP1 domains of Gag [18]. Linking two hexamers were homodimeric interactions between helices 9 of two CA-CTDs consistent with findings that CA-CTD forms a strong dimer interface in the major homology region [19]. CA-CTD also forms intra-hexameric contacts with the CA-CTD of adjacent Gags, but unlike in the mature capsid no large interface between CA-CTD and CA-NTD was observed. This is consistent with data showing CA-CTD domains alone can form into an immature-like lattice, however virus like particles lacking CA-NTD show abnormal size distributions [20]. CA-NTD showed intra-hexameric interactions between helix 4 and helices 5 and 6 of adjacent CA-NTD molecules in agreement with work showing mutations in these regions result in defective virus particles [21]. CA-NTD was also found to form a trimeric interaction with neighboring hexamers, but the cryo-ET density in this region was not as strong as the intra-hexameric contacts. Cryo-EM additionally found structured density in the region of SP1, but no crystal structure of SP1 existed for fitting. Mutations in SP1 regions adjacent to CA-CTD alter the morphology and assembly properties of immature HIV-1 viral particles [22,23].

Cryo-EM experiments have lent much to the understanding of Gag-Gag interactions occurring in the immature HIV-1 lattice, but this work is based on static structures and precludes information related to dynamics. Likewise, TIRF microscopy data has provided new insights into the dynamics and kinetics of HIV-1 viral particle formation, yet cannot resolve the interactions between individual Gag molecules. The plethora of structural and biophysical data on the assembly process make simulation ripe for bridging the gap between static molecular structures and dynamic microscopy data. Previous simulation studies of Gag have primarily involved the mature HIV capsid in which the CA domain forms a fullerene cone containing hexameric and pentameric arrangements of CA [24–29]. These studies, due to the high computational cost, were not able to explore full capsid assembly but suggest the importance of capsid trimers as metastable intermediates in early assembly [24,25]. These trimers coalesce into hexameric (and pentameric) assemblies which subsequently form the closed mature capsid. The recent cryo-EM data however, shows a different arrangement of CA between the immature and the mature forms of the virion, so it is not clear if both forms assemble by the same mechanism. Computational studies of the immature virion have shed light on interactions important in stabilization of the hexameric lattice. Ayton and Voth [30] used a coarse-grained (CG) approach to simulate the entire immature lattice, including a model plasma membrane. They found only sites in the CA-CTD were responsible for maintaining the hexagonal symmetry of the virion, though due to the large system size employed (> 250,000 CG sites) the simulation time was limited to hundreds of nanoseconds. Goh et al, [31] used all-atom molecular dynamics simulations to explore the interactions within a Gag hexamer of hexamers in Rous Sarcoma Virus (RSV). Their simulations showed that the CA-CTD was chiefly involved in inter-hexameric interactions, and the hexameric ring was stabilized by interactions occurring downstream of CA, primarily the SP1 and NC regions of RSV. However, the CA-NTD of RSV showed an arrangement more aligned with that of Mason-Pfizer Monkey Virus [32] rather than HIV-1. In both these works the large system sizes precluded the study of the Gag assembly process–occurring on the order of minutes–which is not accessible using standard simulation techniques.

Given the extent of biophysical data available on assembly of HIV-1 Gag, a dynamic model bridging the short timescales of molecular dynamics with the longer timescales occurring in experiments would greatly aid understanding of the assembly process. In this work we introduce a Brownian Dynamics simulation model containing elementary interactions between Gag molecules, while enabling the study of virion formation taking place on the order of minutes. Our strategy was to model a simple Gag-Gag interaction scheme and test whether the model can recapitulate the existing biophysical data. If conditions were not found, then additional parameters were added to the model. The key experimental observations to match are the structure of the immature Gag lattice, rates of accumulation of Gag, rates of exchange of Gag between the growing puncta and the bulk pool, and the relative size and shape distribution of Gag puncta as a function of the concentration of Gag in the membrane. Within this approach, we find that to obtain a hexameric lattice and assembly growth rates on the order of tens per second requires a binding model in which each Gag molecule contains three non-equivalent binding interfaces. The slow rate of growth and the even slower rate of exchange of Gag, as assayed by photobleaching, restricted the range of values that could be used. Using this binding scheme, we explore whether it is possible for the assembly process to terminate at a fixed number of Gags through kinetics alone or whether another factor, such as curvature of the plasma membrane, must be invoked. Additionally, we probe the possible influence of the HIV-1 genome on initiating assembly by acting as a seed to cluster a small number of Gags. Finally, we derive an analytical model of the Gag assembly process to compliment to the simulation results, finding good agreement.

## Methods

We consider the dynamics and interaction of HIV-1 Gag molecules to study the aggregation process that occurs at the plasma membrane during HIV-1 assembly and budding. Gag aggregation and budding occurs on the time-scale of minutes and involves up to 5000 Gag molecules [13] which is beyond the time and system size scales accessible by atomistic molecular dynamics simulations or standardly used coarse-grain methodologies [30,33]. Here we present a simplified model for HIV-1 Gag aggregation that allows for the simulation of the complete assembly process. We represent a Gag molecule by a circle 2.7 nm in diameter. The diameter was chosen such that the resulting lattice reproduced the 8 nm hexameric spacing observed in cryo-EM. When the Gags are packed in hexameric structure, each molecule contacts three others. Thus the model has three potential interaction faces with other molecules. The modeling will examine, the simplest possibility all three interfaces equal, then only two equal and then all three different. To allow for the asymmetric interactions, each circle has a specified orientation vector. Gag molecules undergo translational and rotational motion on a two-dimensional (2D) surface representative of the plasma membrane. Aggregation and budding are accompanied by an outward curvature of the plasma membrane, but it is not known at what stage of the aggregation process membrane curvature begins to take effect and what influence curvature has on the budding process. As such, we performed simulations both in the presence and absence of parameters designed to mimic membrane curvature.

### Model description

**Fig 1:**
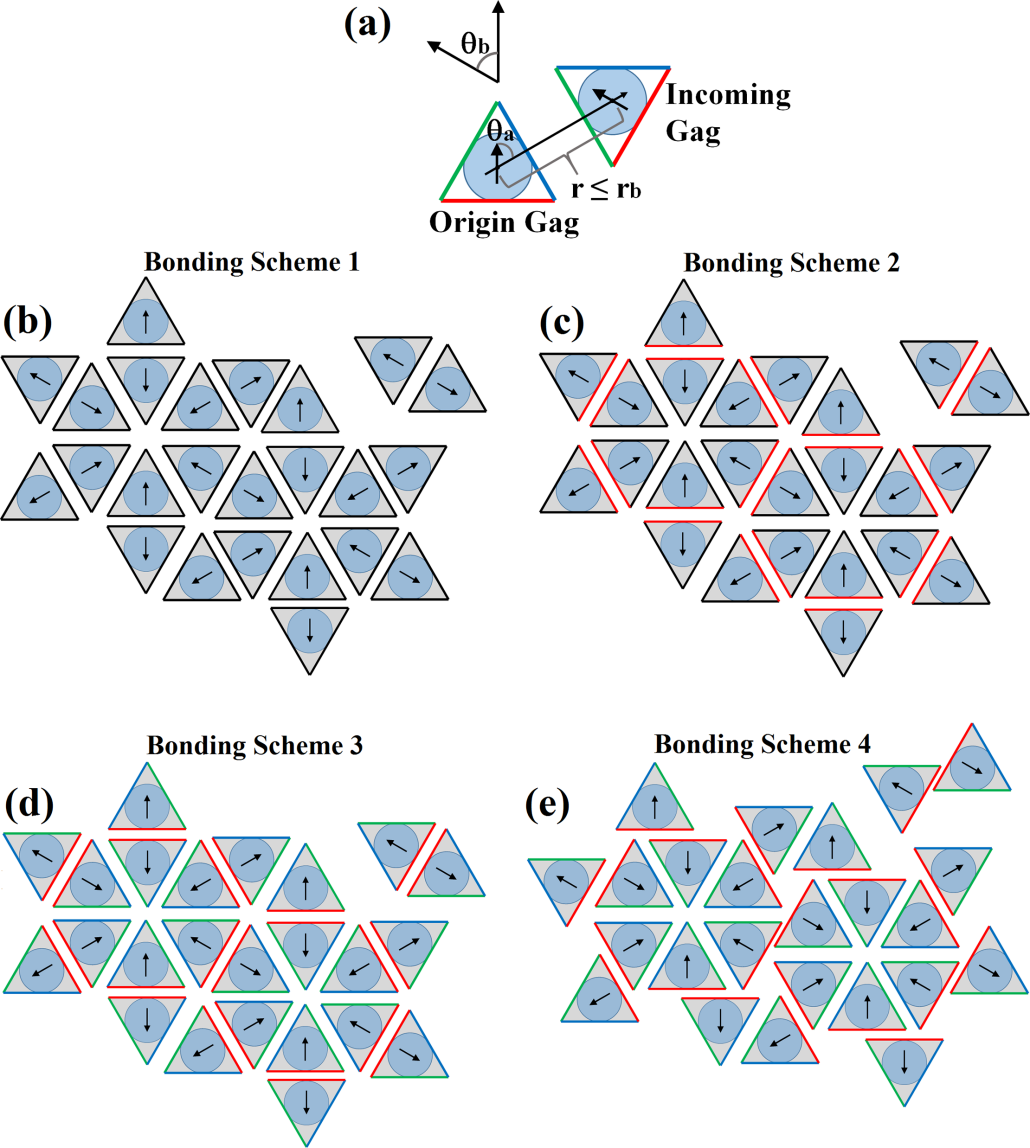
Schematic of the representation of Gag binding in the model. **(a)** Gags are modeled as circular particles with a position and orientation, but in terms of orientation of interaction, they can be thought of as interacting triangles. When two Gags come within a distance r_b_ of each other, a bond can form if 1) The angles of approach (θ_a_) are 60° or 180° or 300° and 2) if the angle between their orientation vectors (θ_b_) is 60° or 180°. **(b)** Bonding between Gag molecules in Bonding Scheme 1. All three faces are equivalent and any face (black) of two Gag molecules can interact. **(c)** Bonding between Gag molecules in Bonding Scheme 2. Two of the three interfaces (black) are equivalent and can interact with each other, and a second dimer interface (red) can only interact with other dimer interfaces. **(d)** Bonding between Gag molecules in Bonding Scheme 3. All three potential bonds are not equivalent. In this scheme, a red interface can only interact with a red interface, and a green interface can only interact with a blue interface. **(e)** Bonding between Gag molecules in Bonding Scheme 4. The available interfaces for Gag-Gag binding is the same as in Bonding Scheme 3, but the orientation of the inter-hexamer bond has shifted relative to Bonding Scheme 3. The bond distances and orientations were taken from the crystal structure 4USN using the center of mass of CA as a reference [16].

Gags undergo translational and rotational motion on a 2D surface. Each Gag molecule is ascribed a position in (x,y) space and an orientation vector. Each individual Gag is permitted to have up to three bonding partners. Three criteria must be satisfied for a bond to form (Fig 1). First, the centers between two Gag molecules must be within a cutoff distance of r_b_. Second, the angle of approach, θ_a_, between two Gags (the angle between Gag orientation vectors and the vector connecting the Gag centers) is equal to ±60 degrees or 180 degrees (plus or minus an angle tolerance parameter, δ_θ_) such that a hexagonal lattice results (simulations with the orientational constraints relaxed yielding spherically symmetric Gag molecules did not result in a hexameric lattice structure). Lastly, the orientation vector between two Gags is θ_b_ = 60° or 180° ± δ_θ_°. Within this simulation construct, a Gag molecule, though still represented as a sphere, could better be thought of as a triangle circumscribing a Gag sphere with binding able to occur when two triangles come within a distance r_b_. Using this model, we varied the interaction between each side of the triangle such that a Gag could have 1, 2, or 3 equivalent faces (Fig 1). Once bound, Gag molecules can unbind at a rate of k_off_, a parameter in the system which is varied over the simulations.

Translational motion of a Gag molecule was allowed to occur stochastically on a 2D surface according to the Brownian Dynamics algorithm [34]:

$$∆\vec{x}=\frac{D\delta t}{k_{B}T}\vec{F}+\vec{S}$$ (1)

where Δx is the displacement of Gag in a given time step δt, D is the diffusion coefficient of a membrane embedded Gag [35], k_B_ is the Boltzmann constant, and T is the temperature. F is the force on a Gag molecule arising from Gag-Gag interactions, and S is a stochastic term with the properties:

$$\langle \vec{S}\rangle =0$$

$${\langle \vec{S}\rangle }^{2}=2D\delta t$$ (2)

Eq 1 was applied to in the x- and y-dimensions modeling Gag diffusion on the 2D plasma membrane. As the average displacement per degree of freedom is $\sqrt{2D\delta t}$, the characteristic force for the system is $F_{c}=\frac{k_{B}T}{\sqrt{2D\delta t}}=30pN$.

When a Gag molecule is unbound, it undergoes translational motion (Eq 1). Additionally, the Gag orientation vector undergoes random rotations sampled from a Gaussian distribution. Translational and rotational motion occur independently of each other. When the distance between centers of two Gags is less than r_b_, a check is performed to determine if the two Gags are in the proper orientation to form a bond conducive to the formation of a hexameric lattice. If the orientation criteria is not satisfied, and the center-to-center distance between two Gags is less than r_b_, Gags experience a repulsive force to prevent Gag overlap.

The bonding interactions between Gags are as follows: Each pair of bound Gags experience a spring force between them of the form:

$$F_{b}={-k}_{b}(r-r_{0})\hat{r}$$ (3)

Where r_0_ is the equilibrium bond distance taken to be equal to r_b_ and k_b_ is a spring constant scaled to F_c_. The spring constant was chosen to be large enough such that two Gags would remain tightly bound, but not so large that displacement of Gags in a given timestep was greater than r_b_/2 (to prevent Gags from passing through one another). Pairs of Gags also experience a torque applied to their orientation vector that attempts to realign the Gag vectors with their original bonded orientations θ_0_.

$$\tau_{b}={-k}_{\theta }(\theta -\theta_{0})\hat{n}$$ (4)

A 3-body force [36], F_φ_, acting on Gag positions is also applied:

$$F_{\varphi }=-k_{\varphi }(\cos\varphi_{ijk}-\cos\varphi_{ijk}^{0})\frac{1}{\left\|{\vec{r}}_{ij}\|\|{\vec{r}}_{kj}\right\|}\frac{\partial }{\partial {\vec{r}}_{\alpha }}({\vec{r}}_{ij}∙{\vec{r}}_{kj})-\frac{{\vec{r}}_{ij}∙{\vec{r}}_{kj}}{2\|{\vec{r}}_{ij}\|\|{\vec{r}}_{kj}\|}\left[\frac{1}{{\left\|{\vec{r}}_{ij}\right\|}^{2}}\frac{\partial }{\partial {\vec{r}}_{\alpha }}{\left\|{\vec{r}}_{ij}\right\|}^{2}+\frac{1}{{\left\|{\vec{r}}_{kj}\right\|}^{2}}\frac{\partial }{\partial {\vec{r}}_{\alpha }}{\left\|{\vec{r}}_{kj}\right\|}^{2}\right]\;\varphi_{ijk}=\frac{\vec{r_{ij}}∙\vec{r_{kj}}}{\left\|\vec{r_{ij}}\|\|\vec{r_{kj}}\right\|}$$ (5)

where k_φ_ is the triplet force constant and φ_0_ is the equilibrium angle between Gag triplet i-j-k. r_ij_ is the vector connecting Gags i and j, and r_kj_ is the vector connecting Gags k and j. Additionally, α runs over each Gag in the the triplet i-j-k. In the case in which only two Gags are bound, F_φ_ is applied to ensure the relative distances and orientations between the two Gags are amenable to binding additional molecules. This is done by positioning an imaginary Gag at a distance of r_b_ and having orientation vector of 120° to the Gag of interest and calculating F_φ_.

The simulation system includes a square membrane patch of 2 μm per side with periodic boundary conditions. Simulations are initialized either with Gags randomly distributed over the membrane or with an initial puncta that is seeded with a fixed number of Gags, the rest randomly distributed in the surrounding space. At the beginning of each simulation time step we determine pairs of Gags that, based on their distance and orientation, satisfy the relevant binding criteria. If a bond forms, a bonding matrix is updated with the type of bond that has formed. Subsequently, all pairs of bonded Gags are checked to see if they will stochastically break according to the relation: Unbinding = k_off_δt. Next, all forces between interacting Gags are calculated, and the new positions of Gags are updated according to Eq 1. In all simulations the time step was taken to be δt = 50 ns. For this value of δt, the average displacement of Gag would be equal to 0.2 nm. This time step was chosen to ensure that the probability of two Gags overlapping was low. All of the parameters for the Brownian Dynamics simulation are given in Table 1.

**Table 1:** Parameters of the Brownian Dynamics simulation.

r_G_	Diameter of a Gag particle	2.7 nm
**D**	Diffusion coefficient	0.2 μ^2^s^-1^ [35]
**δt**	Simulation time step	50 ns
**F_c_**	Characteristic force	30 pN
**r_b_**	Distance for forming a bond	2.7 nm
**r_0_**	Equilibrium bond distance	2.7 nm
**θ_a_**	Angle of approach for bond formation	0°, 46°, 60°, 134°, or 180°
**θ_b_**	Orientation for bond formation	0°, 46°, 60°, 134°, or 180°
**θ_0_**	Equilibrium bond angle	0°, 46°, 60°, 134°, or 180°
**φ_0_**	Equilibrium triplet angle	120°
**δ_θ_**	Angle tolerance	± 1°, ± 3°, or ± 10°
**k_b_**	Bond force constant	1.75 F_c_nm^-1^
**k_θ_**	Angle force constant	5.0 F_c_rad^-1^
**k_φ_**	Triplet force constant	10.0 F_c_nm^-1^
**k_off_**	Bond off rate	10^2^–10^4^ s^-1^

### Bonding schemes

Four different bonding schemes were used in this study corresponding to the triangle representation of Gag shown in Fig 1. In this triangle representation, each Gag can bind to 3 other Gags for a total of 6 different types of bonds. Bonding Scheme 1 treats all bonds as having the same affinity manifest in an equivalent off rate. Bonding Scheme 2 treats one bond specifically as a dimer interface that joins two hexamers (two red faces of Fig 1) with the other two triangle faces equivalent. Thus two different off rates. Bonding Scheme 3 mimics a different affinity for each Gag-Gag interface. In this scheme all bonds have a separate off rate. Bonding Scheme 4 is equivalent to Bonding Scheme 3 in terms of number of bonds, however the bond between Gag hexamers is displaced to more accurately represent the distances and orientations from the Cryo-EM structure of Schur et al. [16]. The orientational differences between Bonding Scheme 3 and Bonding Scheme 4 are shown in Fig 1.

### Neck constriction simulations

All simulations of HIV-1 Gag assembly are performed on a 2D surface for simplicity; however the process of Gag assembly coincides with the formation of a spherical bud at the plasma membrane. Accompanying bud formation is a constriction of the neck region connecting the growing bud to the membrane, restricting the surface area available for adding new Gags to the assembly. To explore how this decrease in available surface area influences growth of a Gag assembly, we perform a series of simulations henceforth referred to as Neck Constriction Simulations. To mimic the budding of an HIV virion into three-dimensional space, for a subset of simulations we perform a series of transformations on the Gag assembly to maintain the assembly interface of a 3D virion. If the assembling virion is treated as a spherical cap of radius R with the height above the membrane plane h (S1 Fig), then the interfacial area of the neck, A_N_, is

$$A_{N}=\pi (2Rh-h^{2})$$ (6)

How Gag assembly influences curvature of the plasma membrane, or vice versa, is not known, but here we make the assumption that there is a linear dependence between the extent which a virion protrudes from the membrane surface (the height of the spherical cap in Eq 6) and the number of Gags in the virion. For a full virion: h = 2R = C_1_N_max_ where C_1_ is a constant and N_max_ is the number of Gags in a completed virion. Solving for C_1_ we can subsequently compute the height of the spherical cap for any number of Gags in an assembly, from which we calculate the interfacial area of the neck (area of the base of the spherical cap) using Eq 6. A new punctum comprised of the number of Gags necessary to fill the interfacial area of the neck is then placed at the location of the old punctum. This is done periodically throughout the simulation. The frequency of transformations is initially set at one per second, and the frequency is modulated such that a transformation is performed when the punctum increases by 10–20 Gags. This would effectively mimic the decrease in available area for Gag addition as a virion buds from the membrane, slowing the rate of Gag addition to the assembly.

### Gag concentration

The concentration of free Gags in the system was clamped at a specific number of Gags (Taken to be 5000 [1250 Gags/μm^2^] unless otherwise specified) independent of the presence or absence of an assembly. This is done by monitoring the number of Gags in an assembly over a period of time. If the assembly increases by N Gags, N Gags are randomly added into the system in regions void of Gags.

### Clustering algorithm

Gags are determined to be in an assembly by iterating over all of the bonded Gags and then performing a distance search to determine nearby bonded Gags. Each found Gag is added to a list until all Gags have been assigned to a cluster. The largest cluster is then taken to be the primary Gag assembly. In this way, we can track at the number of monomers, dimers, trimers, etc. in the system.

### Growth rate calculations

Punctum growth rates are calculated by performing a linear fit to the size of the largest cluster vs. time. In simulations with fast off rates leading to a Gag punctum falling apart, the linear fit is performed with the largest cluster still contains at least 40 Gags. Some simulations with slow off rates result in a plateau in the size of the largest cluster vs. time as a result of all free Gags aggregating into several small clusters and therefore not able to be added to the growing punctum. In such cases, the growth rate calculations were restricted to the linear portion of the largest cluster vs. time plots defined as the time up to which the second largest cluster in the system did not exceed 40 Gags.

### Analytical model

To accompany the simulations, we also fit the data to an analytical model of Gag assembly. The analytical model comprised two different conditions, one for Gag assembly occurring in 2D and one for Gag assembly occurring in 3D. In 2D, the assembly is assumed to be a circular aggregate of Gag while in 3D the assembly is taken to be a spherical cap. Growth of the assembly is assumed to occur through 2D diffusion on a flat membrane. The differential equation for Gag assembly is:

$$\frac{dN(t)}{dt}=k_{G}\times p(N(t))$$ (7)

where N(t) is the number of Gags in an assembly at time t, k_G_ is a lumped parameter for the growth rate of the punctum which incorporates the density of Gags in the system, the rate of diffusion of Gags to the assembly surface, the probability of incorporation of new Gags into the punctum, and the off rate of Gags from the assembly. p(N(t)) is the perimeter of the assembly, which is a function of the number of Gags it contains. For a 2D circular assembly, p = 2πa where a is the radius of the circular assembly. If the area of the assembly is A_c_, then A_c_ = p^2^/4π = αN, where α is the area per Gag molecule. Eq 7 was solved for both the 2D and 3D cases (See Supporting information) and compared to the simulation results. The parameters used for the analytical model are given in Table 2.

**Table 2:** Parameters of the analytical model.

R	Radius of a virion	60 nm
**N_max_**	Maximum number of Gags in a virion	5000
**k_G_**	Virion growth rate	0.02–0.04 nm^-1^s^-1^
**α**	Area per Gag	9.05 nm^2^Gag^-1^

## Results

While we do not parameterize our system to model specific domains of Gag so as to maintain generality, cryo-EM studies show that interactions between Gag molecules primarily occur in the CA and SP1 domains [16,18]. Gag assembles into hexameric structures which aggregate into a lattice with hexameric symmetry (hexamers-of-hexamers). As it is unclear which Gag interfaces present in the cryo-EM data constitute interactions that drive assembly, we proposed four different models for Gag–Gag interactions. In each bonding scheme a Gag molecule has three bonding interfaces able to bind three other Gags. In Bonding Scheme 1, all interfaces are equivalent. Bonding Scheme 2 has two equivalent interfaces and a third differing interface, while all interfaces are different in Bonding Scheme 3. Bonding Scheme 4 is similar to bonding Scheme 3, but with different orientations for the Gag-Gag interfaces. In each case we assessed the bonding scheme by evaluating the resulting lattice structures formed by the assembling Gags to see if a hexamer-of-hexamers arrangement was formed. For each of the different bonding schemes, different rate constants were varied and we measured the growth rates of the puncta to determine if the bonding scheme could reproduce experimentally relevant growth rates (9–10 Gags per second at the maximum), the experimentally observed effects of the concentration of Gag on the rates of growth, and maximal sizes of 5000 molecules. The number of Gags in an assembled virion has been estimated to be between 2500 and 5000 molecules. Measurements at the higher end often came from studies that had virions arrested in assembly with late domain mutations [12]. Thus, they may have allowed time to accumulate greater numbers of Gags. For the following simulations, decreasing the maximum number of Gags to 2500 did not alter any of the fundamental conclusions on which schemes could or could not recapitulate the experimental observations. At most, some of the rate constants decreased linearly with the maximum number of Gag in the final virion. With a satisfactory bonding scheme we explored the influences of Gag interaction with RNA in forming a nucleation point for Gag puncta, the effect of Gag concentration, and the impact of membrane curvature on the rates of puncta assembly.

### Bonding scheme 1

The simplest approach with this model system is to consider all faces of the Gag triangle representation to be equivalent. In this system, any face can form a bond with any other face and there exists only one off rate k_off_. To test the validity of this model, we performed simulations of a random distribution of Gag molecules and observed the resultant structure of a Gag aggregate. To determine if the structure was hexameric, we calculated the orientational distribution between Gags within a cutoff distance r_c_. If Gags are in a hexameric lattice, the angle distributions should peak around 60 and 180 degrees, which is what was observed in the simulation (S2 Fig).

**Fig 2:**
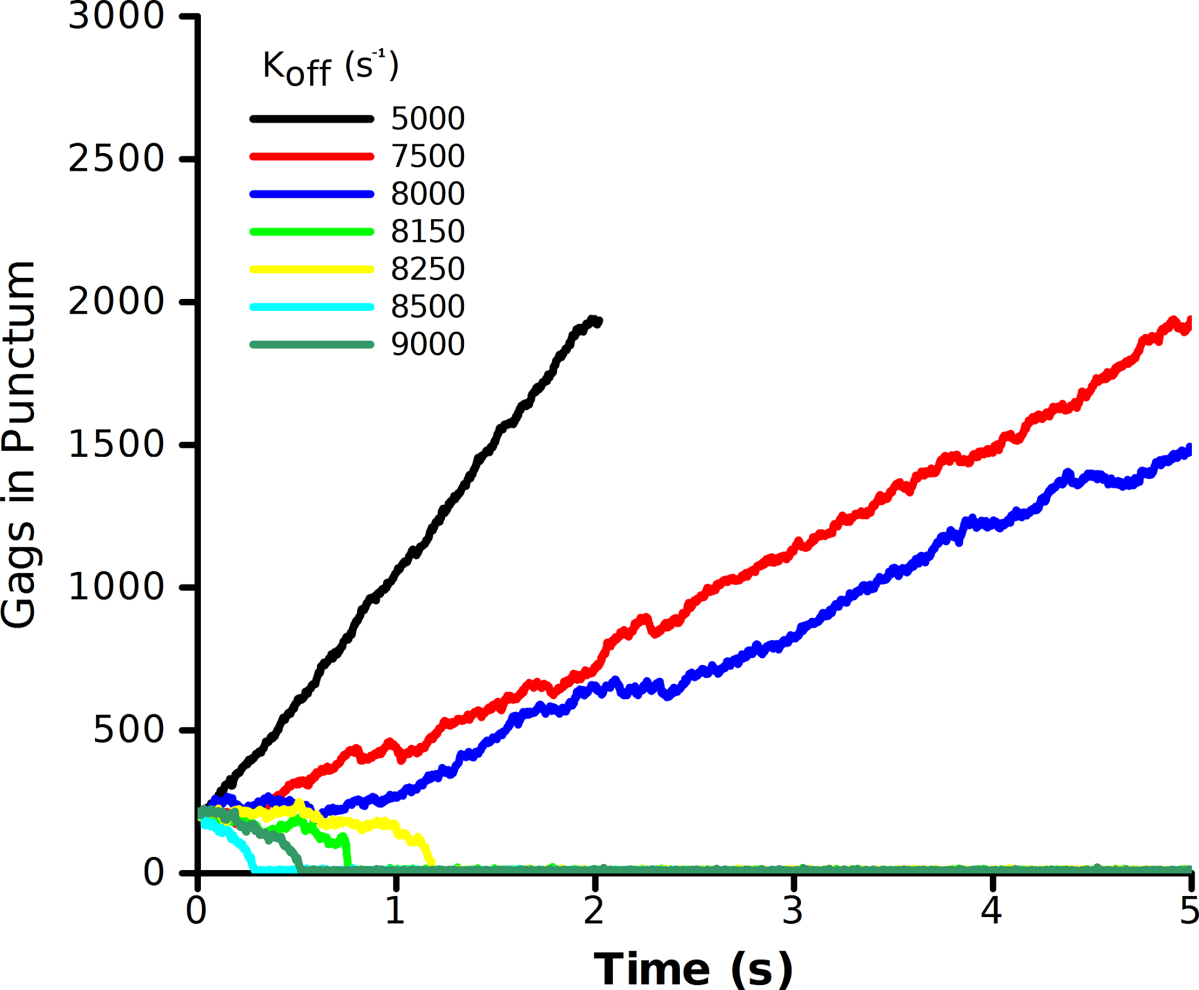
Growth rate of a seeded Gag puncta for Bonding Scheme 1. The simulation consisted of a seeded puncta of 2500 Gag molecules with a background concentration of 5000 Gags at different values of k_off_. Growth rates were determined by a linear fit of the number of Gags in the largest cluster vs. time.

To help limit the ranges of possible Gag-Gag off rates, we started the simulations at the half-way point of maximal assembly with a seeded puncta of 2500 Gags (Fig 2) [Qualitatively similar results were observed if the nascent virion was seeded at the half-way point with 1250 Gags]. The rate of growth of the Gag punctum shows a high degree of sensitivity to the chosen off rate. For a Gag–Gag off rate of 5000 Gags/s, the punctum grows at 900 Gags/s, more than two-orders of magnitude faster than observed experimentally. This rate could be slowed by either increasing the off-rate or decreasing the on-rate. Increasing the off rate to 8000 Gags/s resulted in a growth rate of 156 Gags/s, still much higher than observed. A slight increase of the off rate to 8150 Gags/s caused the seeded puncta of 2500 Gags to fall apart in less than 1 s. The implication of Fig 2 is that Bonding Scheme 1 (all three bonds are equivalent) is not robust; it is highly sensitive to parameter variations. While it is reasonable to assume that the off rate between Gag molecules is not a highly variable parameter, the growth rate of Gags should be reasonably robust to changes in the background concentration of Gag molecules, as is observed experimentally. It has been shown that as the amount of Gag in the membrane increases, the rate of puncta formation decreases from 20 minutes to 4–5 minutes, a four-fold increase in the growth rate [7]. While sensitivity of the puncta growth rate to variation in the off rate parameters does not prove Bonding Scheme 1 to be an inaccurate representation, the lack of robustness seems to suggest that a more complex model is needed to describe experimental observations of puncta growth rates and how those growth rates vary with time and concentration. Bonding Scheme 1 with three equivalent sides produced the correct structure of the Gag lattice (hexamers-of-hexamers), but did not produce biologically relevant rates of puncta growth under conditions that would be robust to small changes in the parameters. Thus, we explored a slightly more complex model.

### Bonding scheme 2

X-ray crystallographic and cryo-EM studies of Gag–Gag interaction indicate that there exists a putative dimer interface between the CA-CTD regions of Gag. We therefore selected one interface of our Gag model to be the ‘dimer’ interface which had different properties than the other two interfaces which we term ‘side’ interfaces. In this iteration of the model, a dimer interface could only bind with another dimer interface and likewise for the two side interfaces.

**Fig 3:**
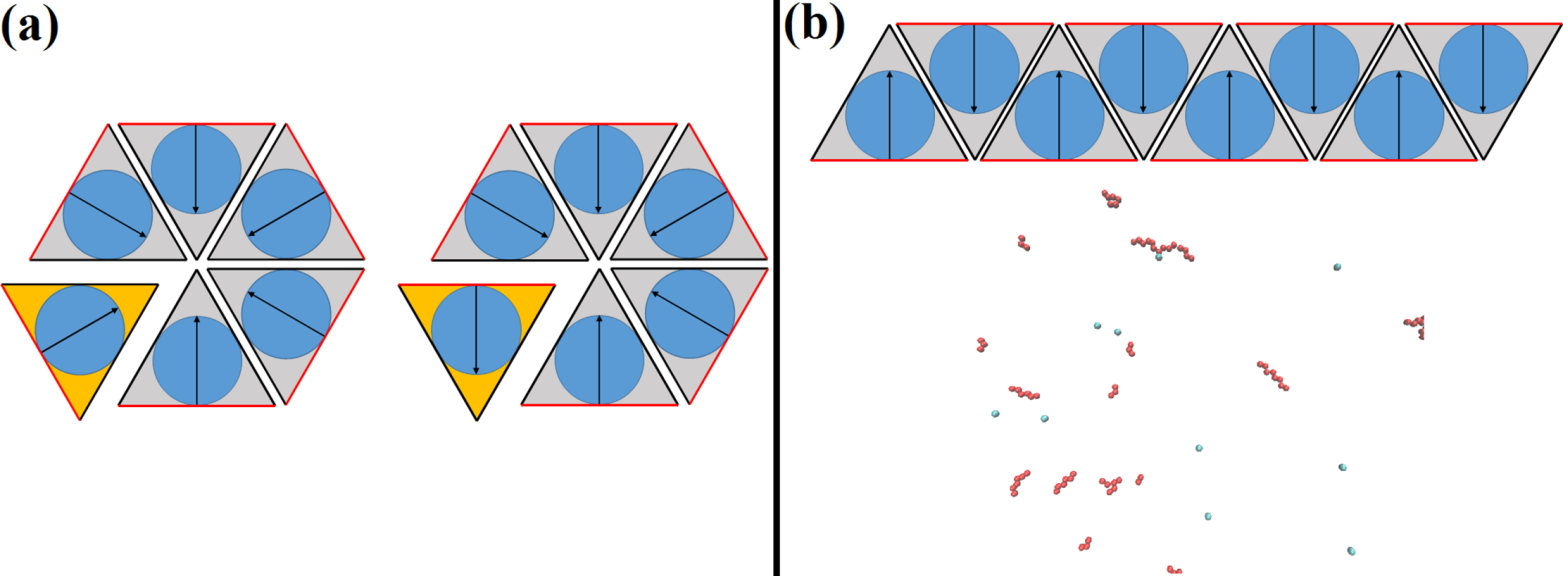
Schematic showing possible bonding scenarios in Bonding Scheme 2. The model consists of one dimer (red) and 2 side (black) interfaces of Gag. **(a)** Bonded Gags are shown in grey and newly arriving Gags shown in orange. (Left) An incoming Gag with its orientation vector pointing towards the center of a forming hexamer resulting in two side-bonds and an outward facing dimer-bond. (Right) Incoming Gag with its orientation vector such that only one side interface can form, leaving a dangling dimer–side interface. In this case, a bond between the dimer and side interface will not form resulting in a less stable hexamer. **(b)** Schematic of the long chain formation found in Bonding Scheme 2 (Top). (Bottom) Example of long chains forming in Bonding Scheme 2.

Simulations of Gags with two equivalent sides and a third, dimer interface resulted in unstable seeded puncta. An initial, seeded hexameric lattice of Gags forms fissures after approximately 0.5 s and nearly dissolves by 1 s (S3 Fig). This result occurred independent of the off rates for either the dimer interface or side interface, with decreasing off rates acting to slow the time to puncta dissolution. Upon closer inspection of the Gag–Gag interactions near puncta fissures, it became apparent that having two equivalent side interfaces allowed Gags to form into hexameric structures, some of which were stable and some unstable. This instability may be the result of the two possible Gag hexamers that could form (Fig 3). In the first (Fig 3A Left), the five bonded Gags (grey) all have their orientations such that the dimer interface (red) forms the edges of the hexamer. An incoming Gag (orange) with the same relative dimer interface orientation could complete the hexamer by forming two side-bonds, increasing the hexamer stability. Another scenario results when an incoming Gag molecule has an orientation such that only one side-bond can form, leaving a side–dimer interface (Fig 3A Right). In this model, interaction between a side interface and a dimer interface will not form a bond causing instability and a fissure in the hexameric structure. A simulation of thousands of Gags interacting over seconds resulted in frequent bond formation that was not conducive to a hexameric lattice structure and resulted in dissolution of seeded Gag puncta. A second complication was the formation of long chains of Gags (Fig 3B), structures that have not been observed experimentally. These factors led us to drop a model with two equivalent faces and, instead, investigate a model in which Gag has 3 non-equivalent bonding faces.

### Bonding scheme 3

We performed simulations of Gag assembly in which each bonding interface was different. This model can form a dimer interface as in cryo-EM structures, and two differing side interfaces, disallowing unstable side interactions that arose in Bonding Scheme 2. Two different values for k_off_ were used, one for the dimer-bond and one for the side-bond. A third off rate differentiating the two side-bonds could be implemented into the model, but as there is no evidence that a left-ward side-bond is any different than a right-ward side-bond, the off rates for the side-bonds were kept equal.

**Fig 4:**
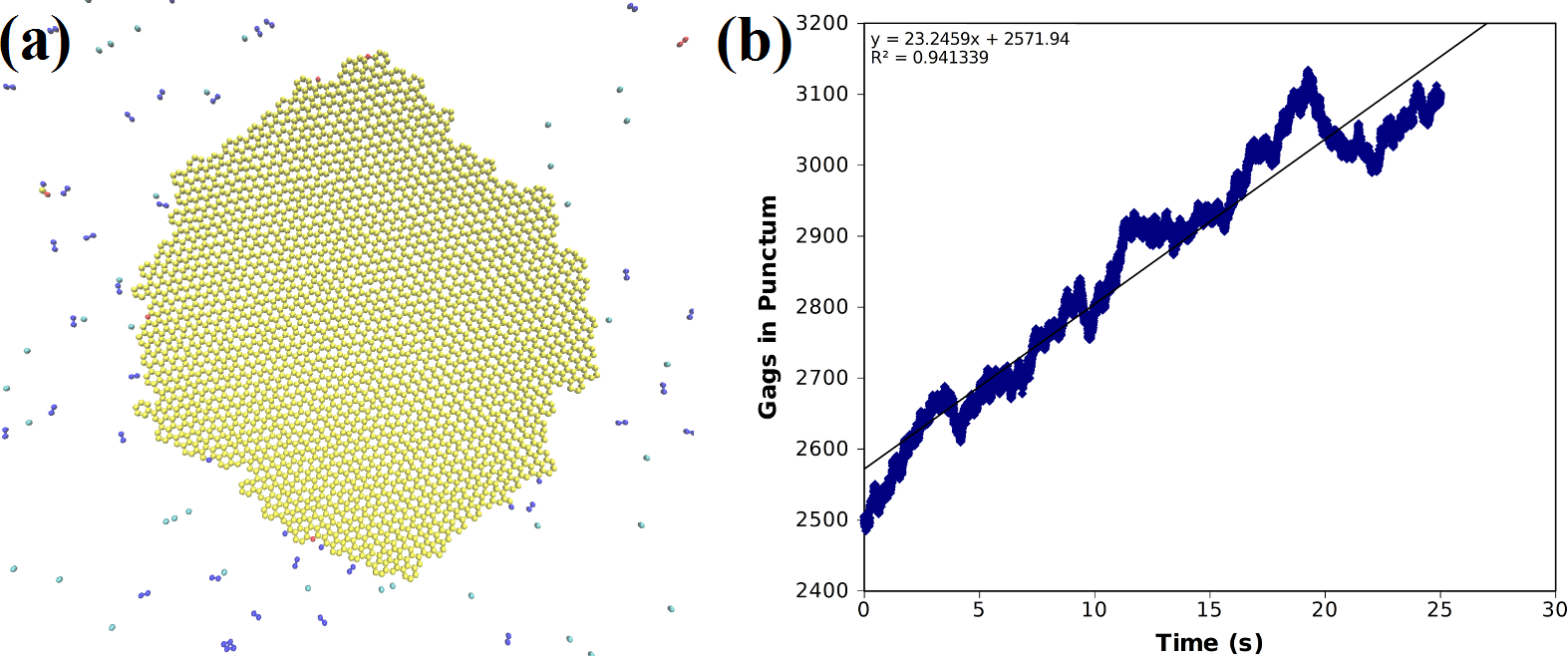
Simulation results for Bonding Scheme 3. **(a)** System snapshot of a seeded puncta of 2500 Gags with 5000 background Gags after 25 s showing the resultant hexameric lattice structure: side-bonding off rate = 1.2x10^4^ Gags/s, dimer-bond off rate = 100 Gags/s. **(b)** The number of Gags in the puncta vs. time. Solid line is a linear fit yielding a growth of 23 Gags/s.

This model initiated with a pre-existing seeded puncta produced a stable hexagonal lattice (Fig 4A). The dimer-dimer interaction is believed to be the most stable, so in most simulations we used a slower off rate for this interaction. We found an wide variety of off-rates for the dimer-dimer and the side-bonds gave biologically relevant growth rates on the order of tens per second and an example of the time course of the growth is given for one simulation of one such asymmetric off-rate for the side bonds of 1.2x10^4^ Gags/s and an off-rate for the dimer-dimer interface of 100 Gag/sec (Fig 4B).

We also measured the radial distribution function (RDF) of all bonded Gags which describes how the Gag density varies around each bonded Gag molecule (S4 Fig). The first peak in the RDF corresponds to the dimer and side-bonds occurring in the system, both having an equilibrium distance of 2.7 nm. A second peak in the RDF corresponds to the second-nearest neighbor bonded Gags, which could belong to the same hexamer or an adjacent hexamer. Together, this indicates that a hexameric lattice was formed. The hexamer to hexamer distance was measured and found to be 8.27 ± 0.18 nm, in good agreement with the cryo-EM hexamer spacing of ~8 nm [37]. Thus, this model reproduced both correct puncta structure and growth rates.

**Fig 5:**
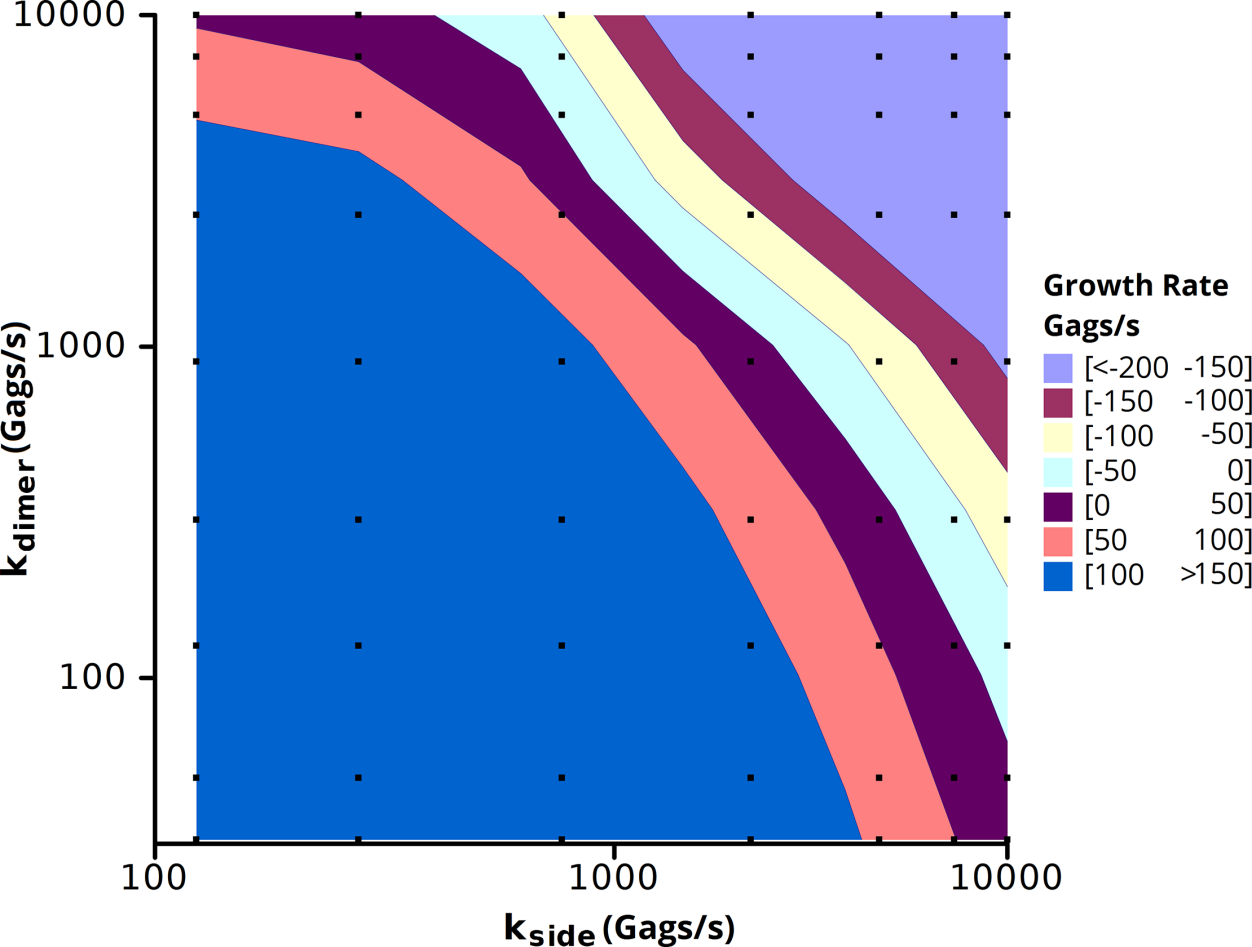
Growth rate contour in Bonding Scheme 3 as a function of side-bond and dimer-bond off rates. Simulations were run for 10 s on a 2.0 μm x 2.0 μm membrane patch with a seeded puncta of 2500 Gags with 5000 background Gags. Growth rates were measured by a linear fit of the number of Gags in the puncta vs. time (S5 Fig). Squares represent the simulations performed. Each data point is the average of 3 independent simulations.

We next explore the space of different off rate constants to determine the biologically relevant regimes and their sensitivity to variations in parameters. We performed simulations on a 2.0 μm x 2.0 μm membrane patch containing a seeded puncta starting with 2500 Gags and a background concentration of 1250 Gags/μm^2^ with varying off rates for the dimer and side-bond interfaces (Fig 5). The contour of growth rates showed two regimes in which this model reproduced biologically relevant growth rates, one with a slow off rate for the side-bond (~500/s) and a fast off rate for the dimer-bond (~5000/s) and one with a fast side-bond off rate (~5000/s) and a range of off rates for the dimer-bond (~100–5000/s). Simulations were also run assuming equivalent to 1250 Gags at the midpoint of assembly (~2500 Gags in the assembled virion) with similar results (S6 Fig).

**Fig 6:**
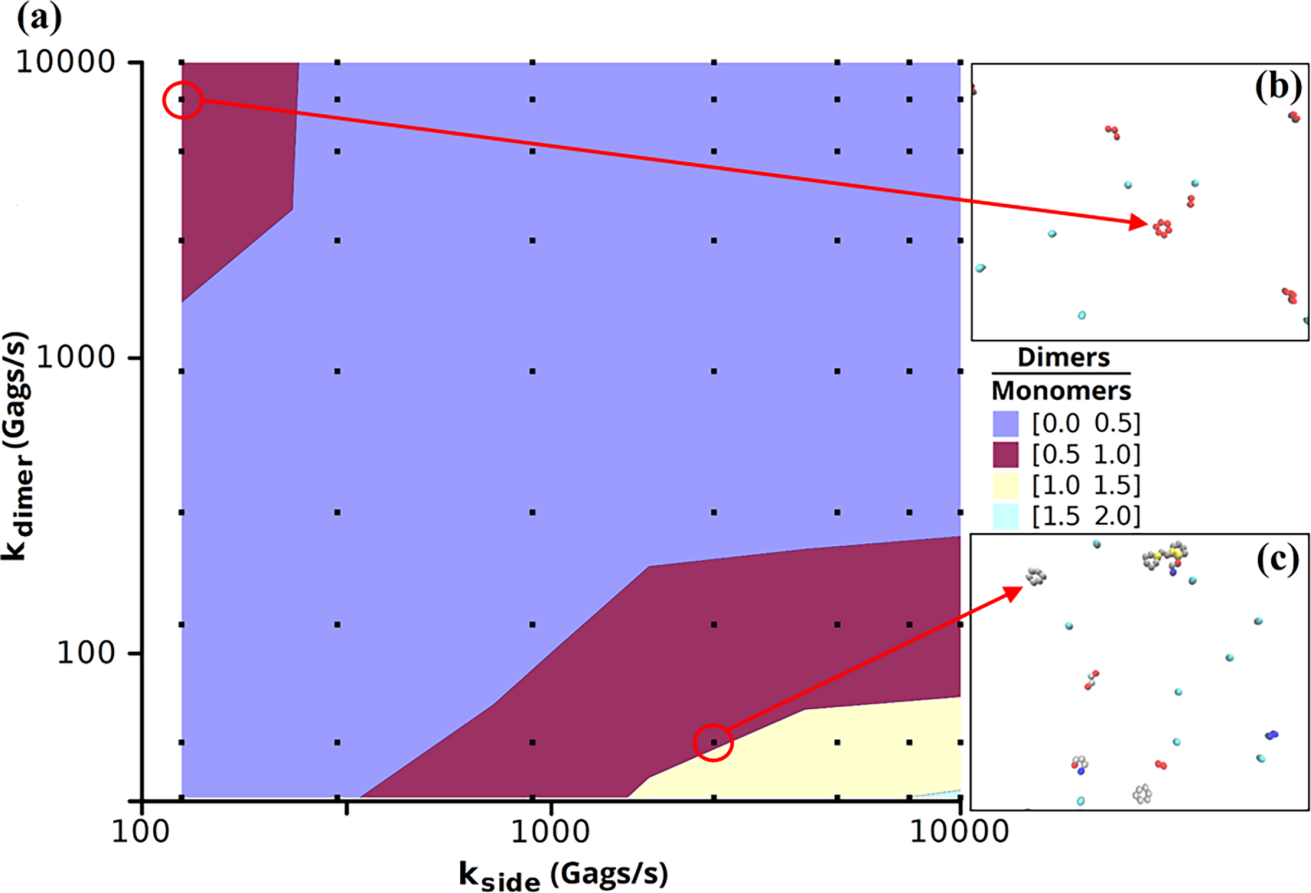
Ratio of dimers to monomers in the simulations using Bonding Scheme 3. **(a)** Dimer monomer ratio as a function of side-bond and dimer-bond off rates. Squares represent the simulations performed. Each data point is the average of 3 independent simulations. **(b)** A hexameric unit in which the hexamer is composed of a six monomers each having a side-bond. **(c)** A hexameric unit in which the hexamer is composed of a trimer-of-dimers.

From observing the growth in Bonding Scheme 3, the side-bonds formed the Gags into hexamers and the dimer-bonds linked the hexamers. Thus, a slow off rate for the side-bonds and a fast off rate for the dimer-bonds resulted in a number of free-floating hexamers which were unable to link together. Oppositely, a slow dimer-bond off rate and a fast side-bond off rate resulted in many Gag dimers which were much less likely to interact to form hexamers (Fig 6). In the simulations in which the off rates for the side-bonds and the dimer-bonds were near equivalent, monomeric Gag dominated the system. When the off rate for side-bonds was increased approximately an order of magnitude greater than the dimer-bond, the relative distribution of monomers to dimers approached 1. Further increasing the off rate of the side-bond relative to the dimer-bond (lower right portion of Fig 6) drove the system into a state with more dimers than monomers. Increasing the off rate of the dimer-bond relative to the side-bond (upper left corner in Fig 6) also resulted in systems with a measureable proportion of Gag dimers, but these systems did not correspond with experimental growth rates (Fig 5). Observing a hexamer with fluorescence microscopy is difficult given the large level of background fluorescence. Experimental studies have not been able to precisely measure the ratio of Gag monomers to dimers *in vivo*, but *in vitro* measurements of Gag CA place the monomer-dimer equilibrium at 18 μM [19] indicating a significant population of Gag dimers. Additionally Kutluay and Bieniasz [3] measured the relative distributions of Gag monomers and multimers both in the cytoplasm and at the membrane of 293T cells finding little multimerization of Gag in the cytoplasm, with multimerization increasing at the plasma membrane. This would seem to suggest that the more relevant regimes in our simulations are those which lead to large populations of both monomers and dimers: when the off rate for the side-bond is a factor of 10–100 fold greater than the off rate for the dimer-bond.

**Fig 7:**
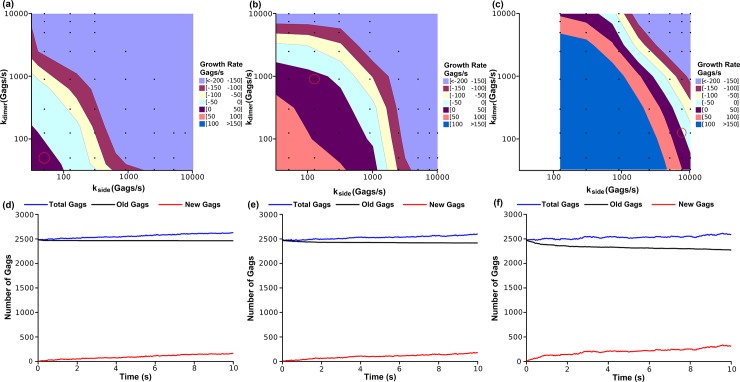
Influence of the binding angle tolerance on the kinetics of puncta assembly in Bonding Scheme 3. Shown are the growth rate contours (Top) and exchange rate plots (Bottom) corresponding to a bonding angle tolerance of δ_θ_ = ±1° (**a**, **d**), δ_θ_ = ±3° (**b**, **e**), or δ_θ_ = ±10° (**c**, **f**). Squares represent the off rates at which simulations were performed. Each data point is the average of 3 independent simulations. Red circles around an individual data point indicate the simulation used to generate the corresponding exchange rate plot. The percentage of original Gags remaining (Old Gags) from a seeded punctum of 2500 Gags was 99% **(d)**, 97% **(e)**, and 91% **(f)**.

When two Gags are within a specified binding distance, certain angle criteria for a bond to form must be met such that a hexagonal lattice eventually results. The allowable amount of variation for meeting these criteria is set by the binding angle tolerance parameter δ_θ_. We tested the effect on growth rate contour of varying the binding angle tolerance δ_θ_ (δ_θ_ = ±1° (Fig 7A), δ_θ_ = ±3° (Fig 7B), and δ_θ_ = ±10° (Fig 7C). A greater tolerance parameter increases the likelihood of bond formation when two Gags are within the bonding distance. Therefore, decreases in δ_θ_ lowers the on rate (defined as the number of Gag-Gag encounters that result in bond formation per unit time), but have no effect on the off rate. Decreasing the bond angle tolerance acted to decrease the off rate required for the Gag puncta increase at a biologically relevant growth rates (~tens per second). The off rates which yielded growth rates of 0–50 Gags/s in Fig 5 were roughly on the order of 10^3^ Gags/s for δ_θ_ = ±10°, 10^2^ Gags/s for a bond angle tolerance of δ_θ_ = ±3° and 10^1^ Gags/sec for δ_θ_ = ±1°.

The growth rate of puncta is dictated by the difference of the on rate to the off rate. The on rate is imposed by the number of bond-forming collisions that occur between Gag molecules. Decreasing the bond angle tolerance decreases the number of bond-forming collisions and therefore a lower off rate is required to maintain growth. Thus, for each of the angle tolerances there is an off rate constant that can give an equivalent growth rate that is observed experimentally. To distinguish between these we examined the predicted rate of exchange of Gag molecules between the puncta and the bulk population. The high angle tolerances, with the higher effective on rate, require a faster off rate and thus a faster exchange of Gags between the growing puncta and the Gags in the surrounding environment. The exchange rate of Gags for puncta growing at a rate of 10–20 Gags/s was calculated for different values of the bond tolerance parameter (Fig 7D–7F). Starting a simulation with a seeded puncta, we measured as a function of time the fraction of Gags remaining from the initial seed and the fraction that were added from the surrounding solution. With a bond angle tolerance of δ_θ_ = ±1°, after 10 s of simulation time 99% of Gags from the initial seed remained in the punctum. This value decreased to 97% for δ_θ_ = ±3° and further to 91% for δ_θ_ = ±10°. From this data, we infer that the simulation can produce biologically relevant growth rates over a wide range of off rates by changing the number of successful bond-forming collisions (by changing δ_θ_). The effect of this is to alter the mechanism by which a growing Gag punctum forms. At the two ends of the spectrum, a punctum may grow either by rarely losing Gags and adding Gags at a very slow rate, or by adding many Gags quickly but also rapidly losing Gags to result in a net increase of tens per second. Fluorescence Recovery After Photobleaching (FRAP) experiments suggest that exchange of Gags between growing puncta and the surrounding environment is minimal [7], but this data was not quantitatively measured. For this reason we performed the majority of the simulations with δ_θ_ = ±10° such that there was only modest exchange of Gags between the punctum and its surroundings, but we note that it is possible for δ_θ_ to take on other values leading to varying exchange rates. An experimental assessment of this value should help narrow down the predicted binding angle of tolerance.

### Influence of RNA

**Fig 8:**
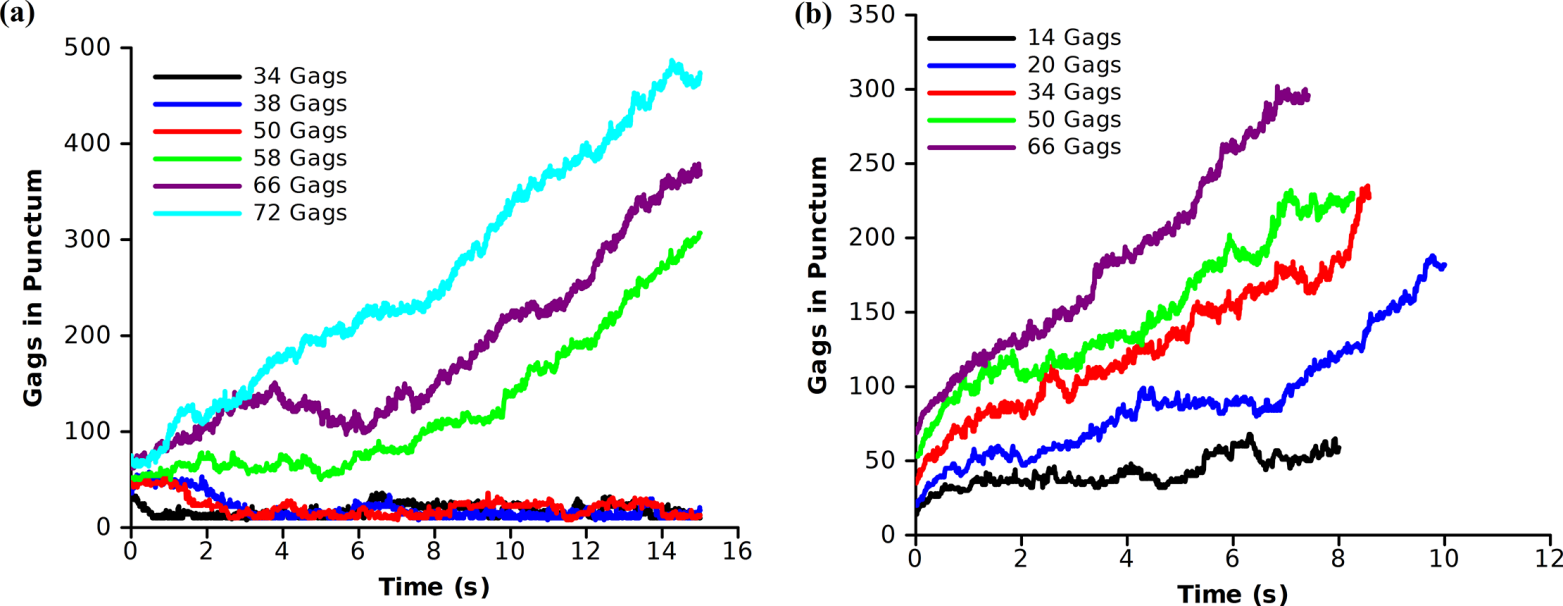
Minimum Gag aggregates for punctum growth. Growth rates of small puncta of varying sizes using a side-bond off rate of 5000 Gags/s and a dimer-bond off rate of 500 Gags/s. Simulations were performed in a 2.0 μm x 2.0 μm with initial Gag seeds of varying sizes and a background concentration of 5000 Gags. **(a)** Gag puncta size as a function of time for varying initial sizes of Gag assemblies. **(b)** Gag puncta size as a function of time for the case in which the initial Gag assemblies were not allowed to unbind, mimicking seeding of a Gag assembly by the HIV genome.

The majority of the growth rate simulations were performed at the midpoint of maximal assembly (1250 or 2500 Gags). We tested if the same off rates would yield biologically relevant growth rates in the early stages of the assembly of a punctum. We selected a biologically relevant growth rate from Fig 5 (side-bond off rate = 5000 Gags/s and dimer-bond off rate = 500 Gags/s) and performed simulations with small initial Gag seed sizes to determine if there is a minimum Gag aggregate that is needed for punctum growth (Fig 8A).

Using the off rates that gave a biologically relevant growth rate, puncta with 50 or fewer Gag molecules were not stable and disassembled within the first 3 s of simulation time. Larger assemblies showed an initial slow growth phase which increased with increasing numbers of Gags in the punctum. Repeated simulations indicated that if a puncta was seeded with fewer than 50 Gags, it would be unlikely to proceed with continued growth. When Gag concentration at the plasma membrane is low, assembly would be an unlikely event. This suggests that another factor may contribute to help initiate coalescence of the Gag molecules at the plasma membrane. A potential clue comes from fluorescence microscopy experiments where it was observed that within minutes after the HIV genome is recruited to the plasma membrane, a Gag punctum assembles at that spot [5]. This raises the possibility that the HIV genome, or an equivalent RNA, acts as a seed to initiate Gag assembly. Further, the genome is believed to arrive at the membrane with a finite number of associated Gags. If the presence of the HIV genome, along with a finite number of Gags, acts as a nucleation point for punctum growth, it could alter the number of Gags necessary to initiate a growing punctum. To replicate this scenario, we performed simulations in which the initial seed of Gags were not allowed to dissociate (zero off rate) such as to mimic the HIV RNA maintaining an aggregate of Gag molecules. Under these conditions, the initial Gag seeds -which are prevented from dissociating- are still able to increase in size (Fig 8B). Thus even when the concentration of Gag at the membrane is low, the HIV genome, or an equivalent RNA, can act as a seed to locally concentrate Gags and encourage virion formation.

### Steady state simulations

Experimental data show that the result of the aggregation process is a virion with roughly 2500–5000 Gags [12]. TIRF microscopy studies show that following transfection the concentration of Gags at the plasma membrane continually increases over time yielding faster times for complete puncta formation, yet the maximum fluorescence for any given puncta remains relatively constant [7]. This suggests that at completion, each viral bud contains approximately the same number of Gag molecules. The mechanism by which HIV maintains a consistent number of Gags per virion is not known. Here we investigate if it is possible for the number of Gags per puncta to plateau solely through the kinetics of Gag–Gag interactions. Using the growth rate contour of off rates from Bonding Scheme 3, we selected regions which result in biologically relevant growth rates (~tens per second) and determine the background concentration of Gags which results in zero growth at a fixed puncta size of 2500 or 5000 Gags. To approximate the background concentration of Gags which would lead to a steady state puncta size, we ran simulations of a seeded puncta of 5000 Gags with varying Gag background concentrations for 5 s and measured the growth rates. The growth rates were linearly fit to the background Gag concentration and interpolated to a zero growth rate (S7 and S8 Figs). Simulations were then performed for longer times at these concentrations.

**Fig 9:**
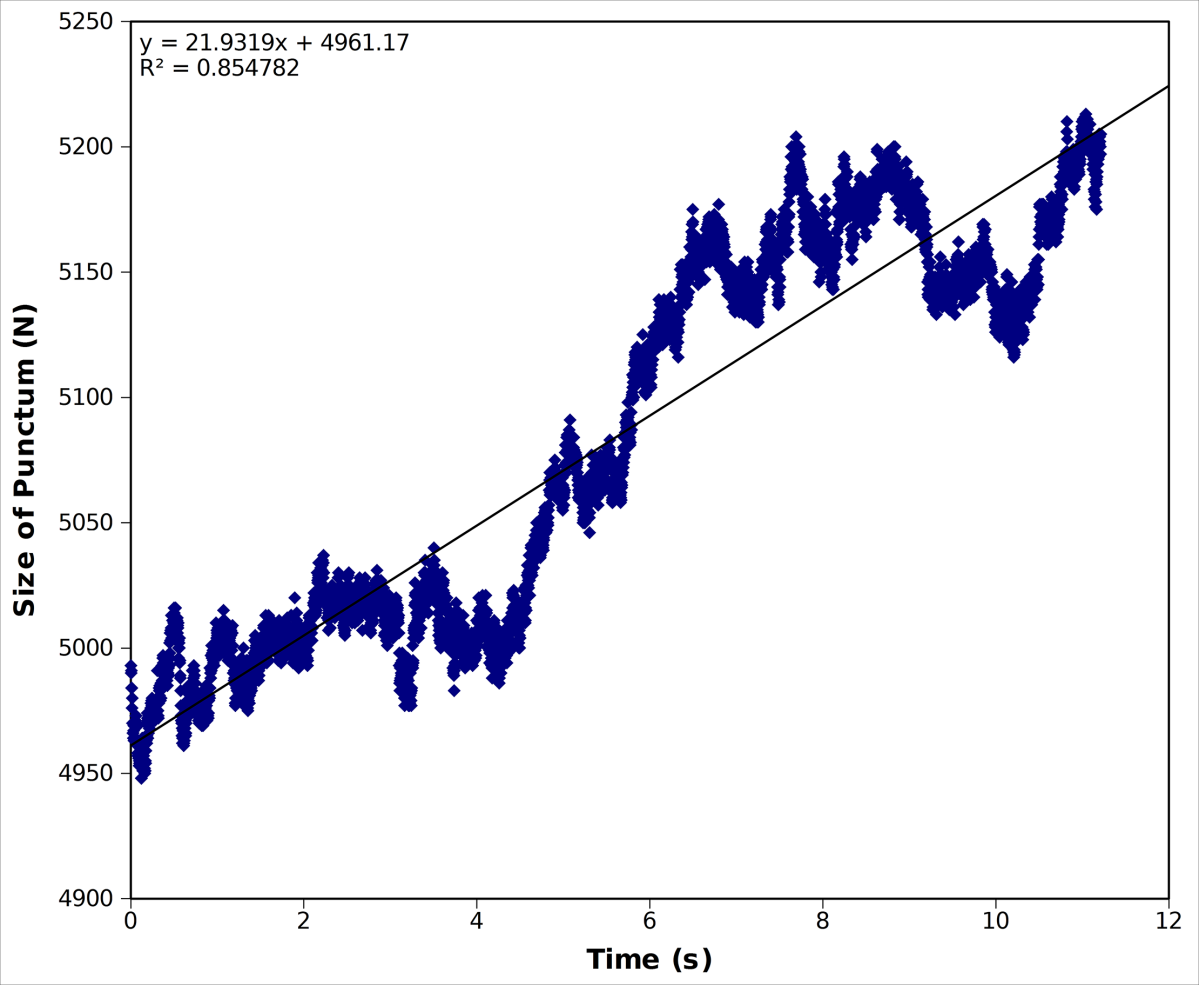
Simulations of puncta at a steady-state. Results of the steady-state simulations starting from a completed punctum of 5000 Gags and a background concentration of 6578 Gags, the background concentration interpolated to produce a zero growth rate. Side-bond off rate = 10^4^ Gags/s, dimer-bond off rate = 2x10^2^ Gags/s.

The result of one steady-state simulation with a side-bond off rate of 10^4^ Gags/s and a dimer-bond off rate of 2x10^2^ Gags/s is shown in Fig 9. Simulations with varying background concentrations predicted 6578 background Gags resulting in a zero growth rate. When a seeded punctum of 5000 Gags was placed with a background of 6578 Gags however, the result was a steady punctum for ~4 s followed by an increase in the growth rate and thus punctum size (Fig 9). The results of the steady state simulations suggest that there does not exist a purely kinetic factor which would cause punctum growth to halt at roughly 5000 Gags. For each combination of relevant off rates, one can find a background concentration for which a punctum containing 5000 Gags would be at a steady state (the rate of addition of new Gags is balanced by the off rate). However, when more Gags are added into the system the steady state balance is shifted, and a new steady state punctum is obtained containing a greater number of Gags, effectively an unstable equilibrium. This conflicts with biological data which finds increasing Gag concentration at the plasma membrane, yet virions are still nearly the same size and contain approximately the same number of Gag molecules. For this reason, it is likely that some other factor contributes to Gag reaching a steady-state value.

### Neck constriction simulations

One possible factor leading to the cessation of assembly is the membrane curvature associated with an assembling virion. At some point the Gag and membrane of the nascent virion must bud outward and, as the neck narrows, the interface where Gag is being recruited to the growing virion decreases in size thereby slowing growth. Further narrowing, all the way down for scission, halts addition of Gags to the virion. To test this hypothesis, we performed simulations mimicking the decrease in the available surface for Gag addition as a punctum grows in size. To simulate the outward bending of the plasma membrane and budding of a nascent virion in our 2D system, we applied a transformation throughout Gag assembly to restrain the size of the punctum to the interfacial area which a 3D virion connects the plasma membrane. This was done by periodically changing the size of the punctum during the simulations. The total number of Gags in the punctum was used to model the punctum as a spherical cap, and the interfacial area of the spherical cap with a flat membrane was computed. A new circular punctum with the number of Gags for the proper interfacial area was then re-seeded in place of the original punctum, and this was repeated throughout the simulation. The result is that when puncta are small, the surface area of a spherical cap and its interfacial area are similar. When a budding punctum reaches 2500 Gags, the result is a hemisphere with an interfacial area equivalent to a circular punctum of 1190 Gags. When the budding punctum has greater than 2500 Gags, the interfacial area decreases, shrinking to zero as the number of Gags in the budding punctum nears 5000.

**Fig 10:**
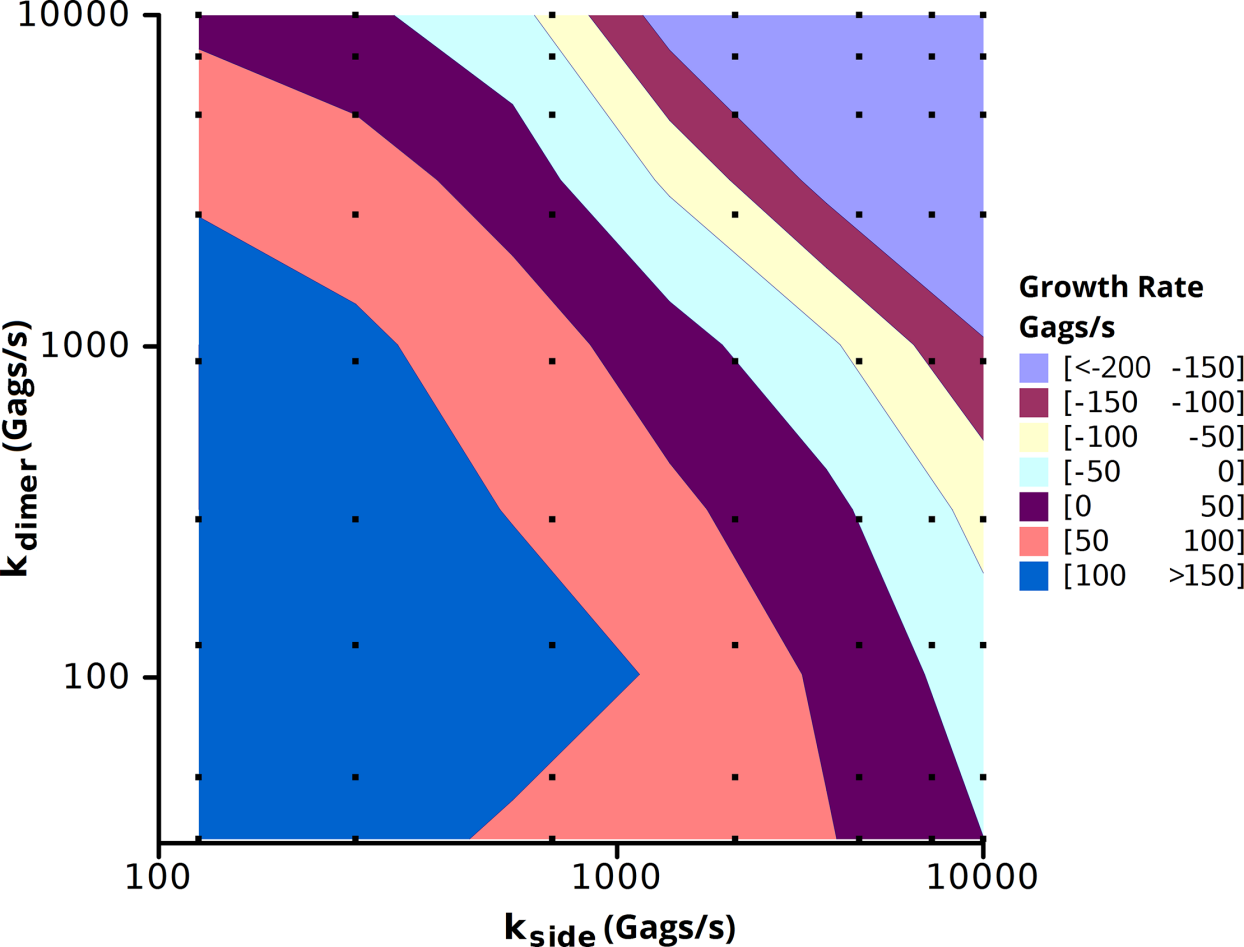
Growth rates for neck constriction simulations. Growth rate contour of the punctum growth rate in the 3 interface model while accounting for neck constriction. Simulations were run for 10 s on a 2.0 μm x 2.0 μm membrane patch with a seeded puncta of 1190 Gags with 5000 background Gags. Growth rates were measured by a linear fit of the number of Gags in the puncta vs. time. Black squares represent the simulations performed. Each data point is the average of 3 independent simulations.

**Fig 11:**
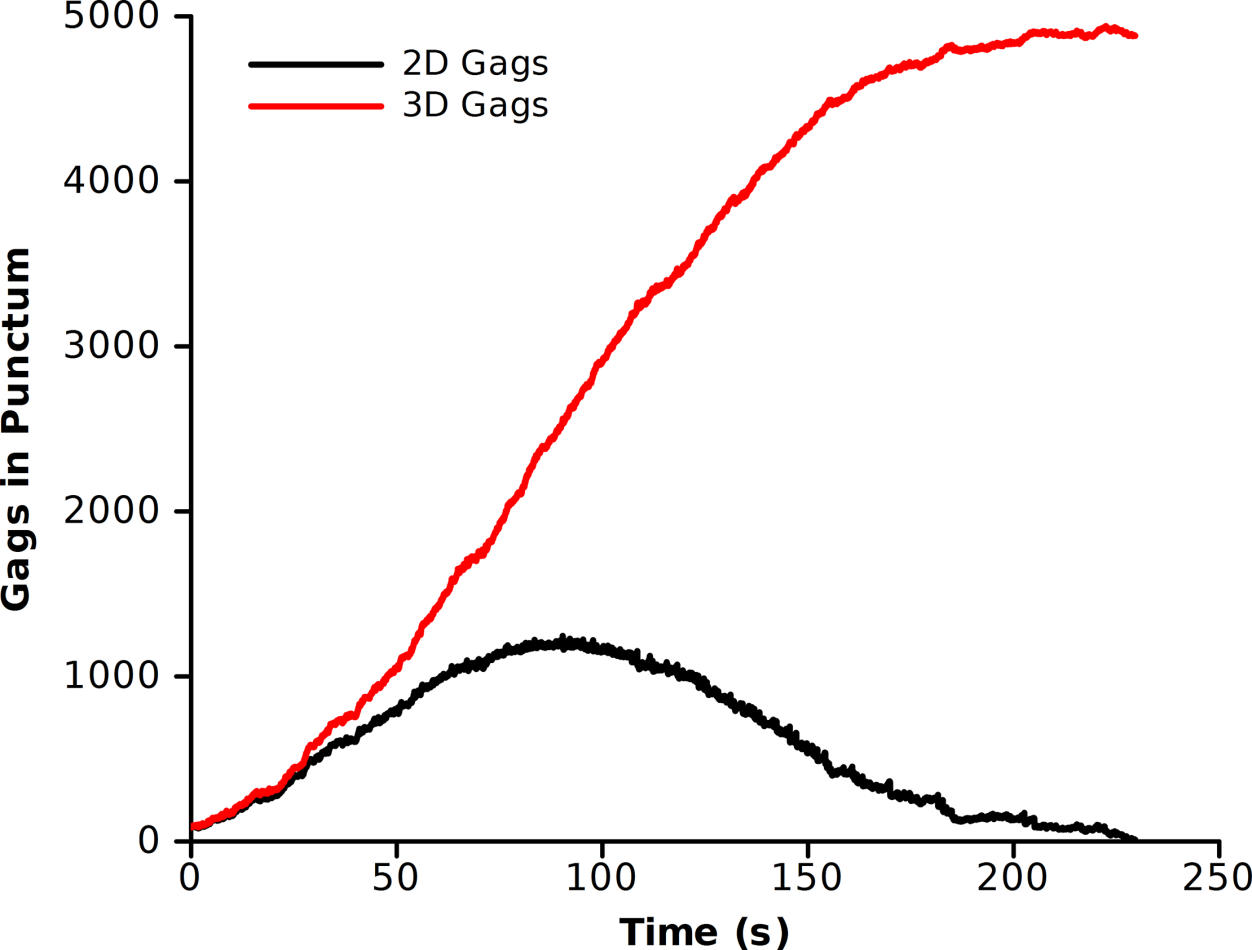
Completion of growth under neck constriction simulations. Growth rates using the resize algorithm with a side-bond off rate of 7500 Gags/s and a dimer-bond off rate of 55 Gags/s with an initial seed of 88 Gags and a background concentration of 5000 Gags. The two different curves indicate the number of Gags in the interfacial area (2D Gags) and the total number of Gags in the punctum, assuming a spherical cap lined with Gags (3D Gags).

Resizing the punctum alters the midpoint of the growth simulations of Fig 5, as the midpoint of virion growth (2500 Gags) now has an interfacial area equivalent to a circular punctum of 1190 Gags. As such, we repeated the simulations in Fig 5 varying the off rates of both the side-bond and dimer-bond to measure the growth rate at 1190 Gags and a background concentration of 5000 Gags (Fig 10) [If it is assumed that the maximum number of Gags is 2500, the results scale linearly]. Comparing the neck constriction simulations (Fig 10) to the original geometry (Fig 5), we find a similar trend in the growth rates, but in the neck constriction simulation, to reach the equivalent growth rate, a slower off rate was required. In other words, with the same rate constants, the growth rate is slower in the neck constriction simulation. This is anticipated as the growth of a punctum would be expected to slow at the interface between the puncta and the cell, and thus the ability to recruit Gag decreases. Longer simulations used the resizing algorithm starting from an initial seed of 88 Gags (Fig 11). The plot shows both the number of Gags in the 3D budding punctum and the equivalent number of Gags for creating an interface of the proper size with the plasma membrane. The Gags in the punctum is a running tally of the net number of Gags that have been added to the punctum since the onset of the simulation, corrected for resizing. This is equivalent to the total number of Gags which line the spherical cap of a budding virion. When the punctum is small, there is little difference between the numbers of Gags in the punctum and the number in the simulated system (the assembly only marginally buds from the membrane surface and thus the interfacial area is similar to the curved surface area). At large punctum sizes (nearing 5000 Gags) the number of Gags in the system differs greatly from the number of simulated Gags in the system. This is due to the fact that as the virion is nearing completion, the area of contact with the membrane (and thus the available area where new Gags molecules can add) has decreased. In Fig 11, the punctum reaches 5000 Gags after 250 s for a growth rate of ~22 Gags/s. This is faster than the typical experimental value for punctum formation (~9 Gags/s) but still within the range of experimental values [7]. This suggests that budding of the virion from the plasma membrane could be the factor that results in a slowing of Gag incorporation at an assembly site such that virions generally retain the same size and number of Gag molecules independent of Gag concentration in the cell and the time taken to complete assembly.

### Bonding scheme 4

**Fig 12:**
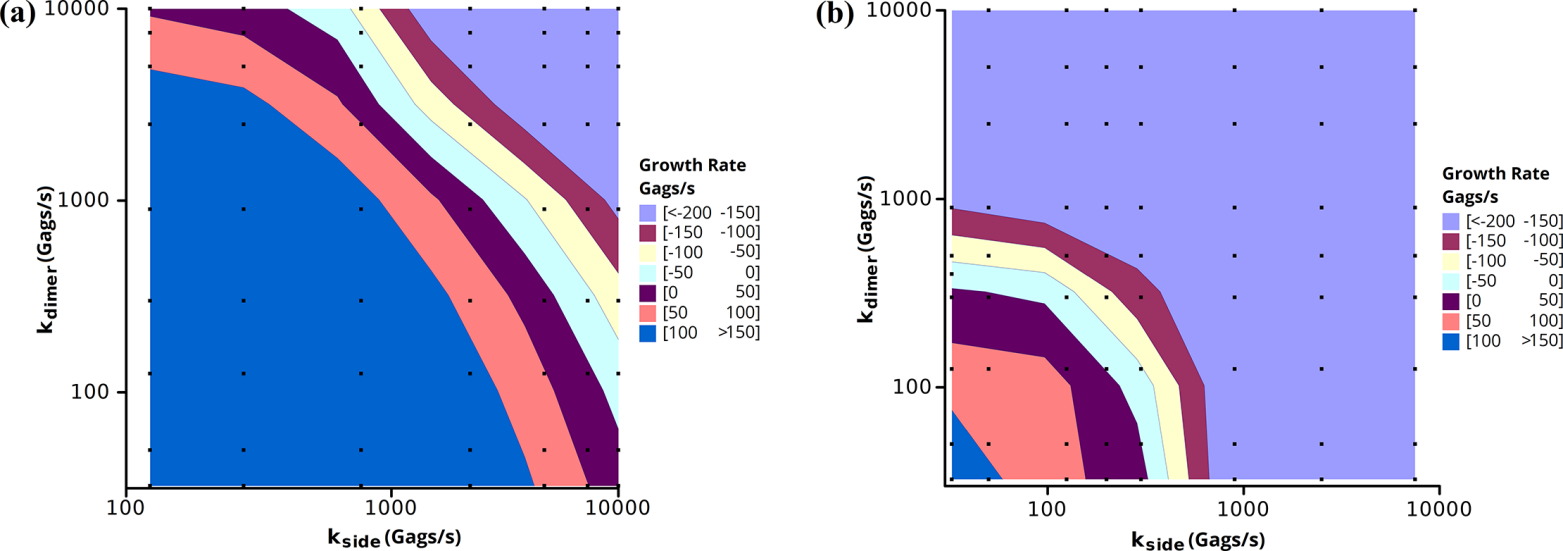
Comparison of the puncta growth rates between Bonding Scheme 3 and Bonding Scheme 4. Simulations were run for 10 s on a 2.0 μm x 2.0 μm membrane patch with a seeded puncta of 2500 Gags with 5000 background Gags. Growth rates were measured by a linear fit of the number of Gags in the puncta vs. time (S5 Fig). Squares represent the simulations performed. Each data point is the average of 3 independent simulations. **(a)** Puncta growth rates using Bonding Scheme 3, the same as in Fig 5. **(b)** Puncta growth rates in Bonding Scheme 4. Note the scale change on the x-axis.

To compare the influence of hexamer packing on puncta growth rates, we altered the inter-hexamer orientation from Bonding Scheme 3 to give Bonding Scheme 4 (Fig 1). This bonding scheme more faithfully recapitulates the Gag-Gag orientations from Schur et al. [18] while still maintaining the 8 nm hexameric spacing. The result of altering the inter-hexamer orientation is to shift the biologically relevant puncta growth rates (10’s of Gags/s) to lower Gag-Gag off rates (stronger Gag-Gag interactions strengths). The comparison of puncta growth rates as a function of Gag-Gag off rates is shown in Fig 12. Interestingly, even though the orientations of the dimer bond were altered in Bonding Scheme 4, both the off rates for the dimer-bond and the side-bonds decreased proportionally from an off rate of thousands of Gags per second in Bonding Scheme 3 to an off rate of hundreds of Gags per second in Bonding Scheme 4. As a results, though the alteration of inter-hexamer orientation alter the off rates at which biologically relevant puncta grow, the trends from Bonding Scheme still hold.

### Analytical model

**Fig 13:**
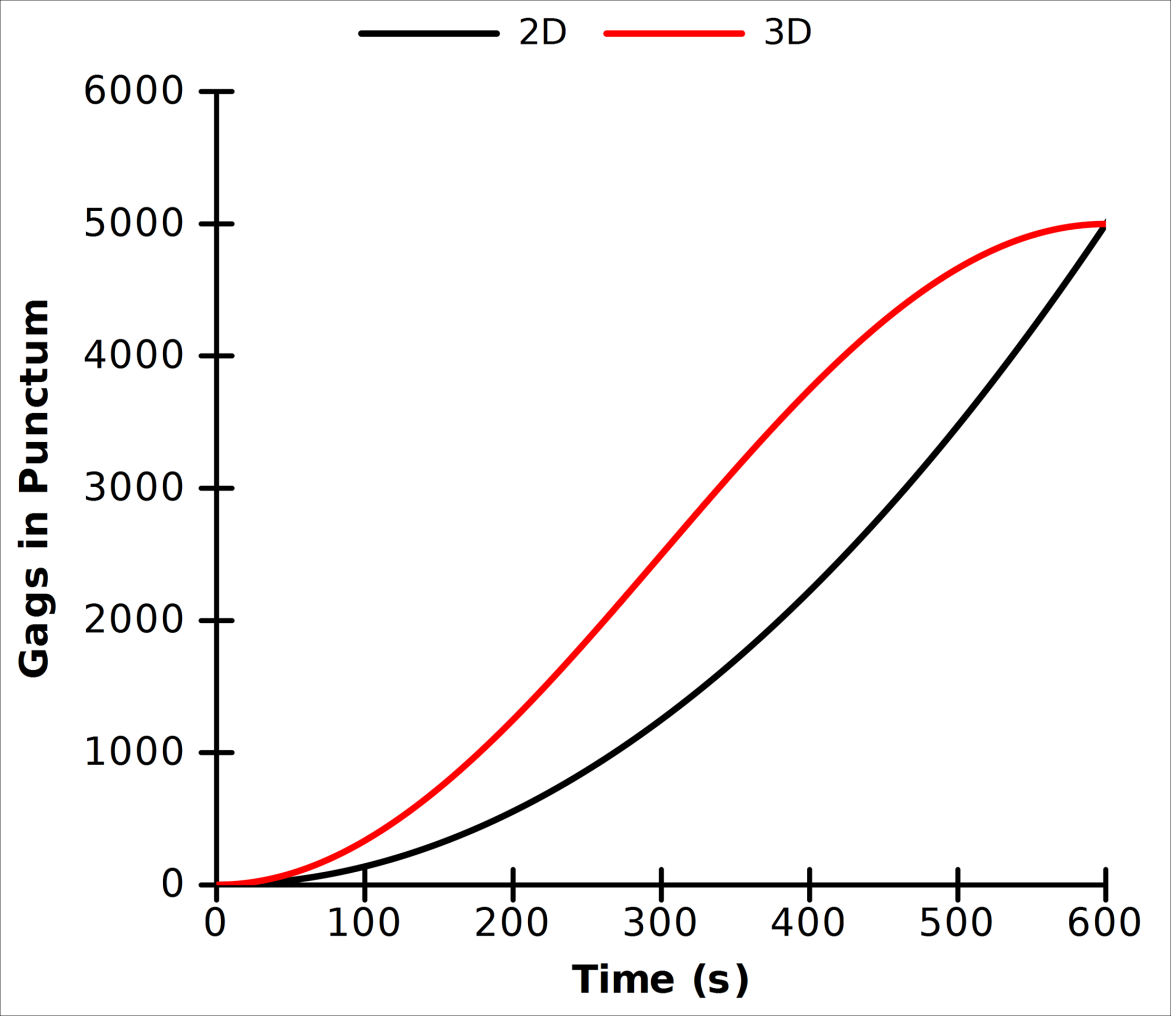
Analytical model for Gag assembly in 2D (Black) and 3D (Red). Curves are generated using the solution to Eq 7 with k_G_ = 0.0221 (Black) and with k_G_ = 0.0347 (Red) so as to reach a punctum comprised of 5000 Gags in 10 minutes.

As a check for the validity of the simulations, we formulated a mathematical model for the assembly of Gag on a 2D surface, and in 3D, similar to the neck constriction simulations. The 2D assembly is taken to be a perfect circle and the 3D assembly to be that of a sphere. Gag molecules are assumed to be recruited to the site of assembly through diffusion in the plane of the membrane. The net number of Gags which are added to an assembly over a given time is related to the on rate (encompassing diffusion to the punctum surface and binding in the correct orientation) and the rate at which Gags leave the assembly, both of which are a function of the length of the perimeter of the assembly. For simplicity, the on rate and the off rate are lumped into a single parameter k_G_ as given in Eq 7. The solutions for both the 2D and 3D cases are shown with the growth rate parameter k_G_ chosen such that a punctum of 5000 Gags forms in 10 minutes (Fig 13). For the 2D curve, the number of Gags in the punctum is related to the square of the time, which is a function that increases without bound. This may explain why there is no kinetic steady state at 5000 Gags in the 2D simulations assuming a constant background pool of Gags (S9 Fig). Conversely, the functional form for the number of Gags in a 3D punctum is sin^2^, which has a maximum, in this case, at 5000 Gags. Of note, the growth parameter k_G_ is 0.0221 nm^-1^s^-1^ for the 2D case and 0.0347 nm^-1^s^-1^ for the 3D case. The growth rate for the 3D case must be 57% faster than in the 2D case to account for the decreased area through which Gags can be exchanged. This is consistent with the simulations where slower off rates (increased k_G_) were needed to achieve physiologically relevant growth rates compared to the 2D case (compare Fig 5 and Fig 10).

The analytical growth rates can be compared to the simulations by comparing the inverse of the punctum perimeter multiplied by the rate of growth of the punctum:

$$k_{G}=\frac{1}{p}\frac{dN(t)}{dt}$$ (8)

where p is the perimeter of the punctum in the simulation. For the neck constriction simulation shown in Fig 11 the growth parameter calculated using Eq 8 is twice that calculated using the analytical model, Fig 13 (k_G_ = 0.071 nm^-1^s^-1^ vs k_G_ = 0.035 nm^-1^s^-1^). The difference is due to the fact that the off rates in the simulation of Fig 11 are such that a punctum of 5000 Gags is formed in a little over 4 minutes whereas the computation in Fig 13 is calculated to form a punctum in 10 minutes. Solving Eq 8 using k_G_ = 0.071 nm^-1^s^-1^ results in a punctum of 5000 Gags forming in 293 s.

## Discussion

In this work we introduced a computational model for the assembly of HIV-1 Gag into immature virions, a process that takes place on the timescale of minutes. The model was built using information about the functional interfaces implicated in Gag assembly, primarily interactions involving CA-NTD and CA-CTD, but was not limited to specific Gag-Gag interactions to maintain generality. Gag was modeled as an equilateral triangle with three possible bonding interfaces. We first set out to determine which bonding schemes, if any, were compatible with experimental data. Comparing the simulation results with the cryo-EM lattice structure of immature Gag and the time course for Gag assembly, we were able to narrow the model down to two non-equivalent side interfaces interacting to form Gags into a hexamer and a single dimer interface acting to link hexamers together. In this way, the model was able to assemble Gag molecules into a hexameric structure with the correct lattice spacing and reproduce biologically relevant punctum growth rates. This functional model was then employed to explore the effect of HIV genomic RNA seeding punctum growth, the presence or absence of a kinetic steady-state, and the influence of curvature of the plasma membrane on the rate of Gag assembly. In order to further corroborate the results of our simulation model, we proposed an analytic model for Gag assembly. Resulting calculations proved complementary and in good agreement with the simulation results.

**Fig 14:**
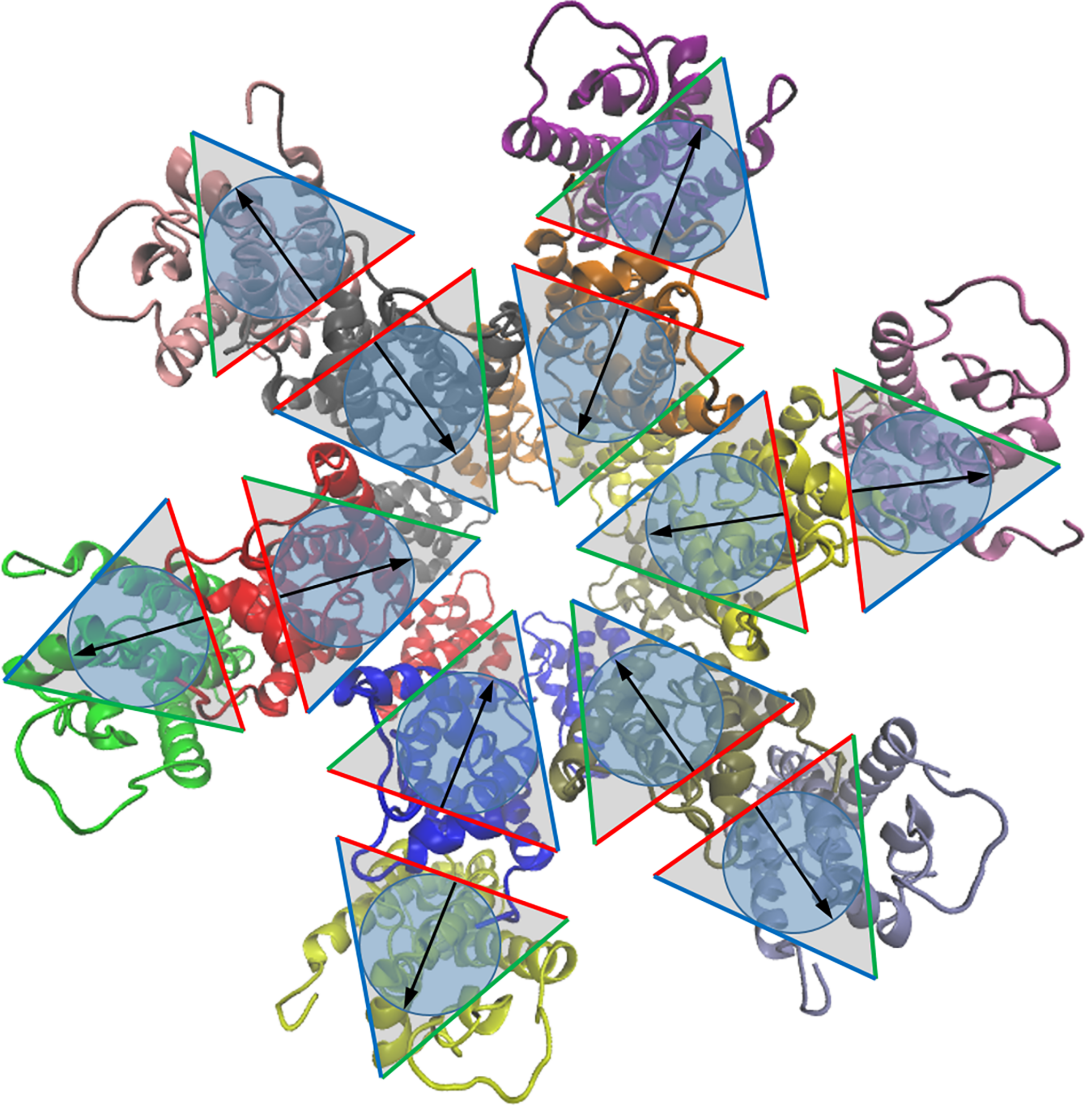
Comparison of cryo-EM structure to simulation model. Cryo-EM structure of Gag CA (PDB: 4USN [16]) looking down from the CA-NTD to the CA-CTD, with the model representation overlaying each Gag monomer.

Evaluating different bonding schemes, the models with all interfaces equivalent or with two interfaces equivalent were not able to recapitulate the experimental data with regards to the growth rate of the assembly and the formation of a regular hexameric lattice. Bonding Scheme 1 with three equivalent bonding interfaces yielded growth rates that were very sensitive to the Gag-Gag off rate, whereas Bonding Scheme 2 with two equivalent side interfaces and a dimer interface resulted in unstable puncta that quickly fell apart and produced long chains of Gags rather than a packed punctum. Bonding Scheme 3 produced a hexameric lattice comparable to cryo-EM data and grew at biologically relevant growth rates. While it is difficult to relate the aspects of Bonding Scheme 3 directly to the available structural data, we can make some inferences from the cryo-EM data. Fig 14 shows the crystal structure of CA fit into the cryo-EM data for the immature HIV-1 Gag lattice along with an overlain representation of Bonding Scheme 3. In this scheme there are two side-bonds that act to induce Gags to form into hexamers. In the model the two side-bonds have the same off rate, but they are not equivalent as they depend on orientation (A blue interface may only bond with a green interface, Fig 14). From the structural data, within a hexamer helix 4 of CA-NTD interacts with helices 5 and 6 of a neighboring CA-NTD, and intra-hexameric interactions between adjacent CA-CTDs are thought to add stability [38]. SP1 forms a six-helix bundle with neighboring Gag molecules [18, 39]. This supports the notion of two side interfaces acting to form Gags into a hexameric shape. Between hexamers CA-NTD has a dimeric interface between helices 1 and a trimeric interface between helices 3 while CA-CTD forms a dimeric interface between helices 9 [16]. The inter-hexameric interactions being different from the intra-hexameric interactions, suggests that the dimer-bond linking hexamers together is fundamentally different from the side-bonds causing hexamers to form. This further supports a bonding scheme with three non-equivalent bonding interfaces. Comparing the effect of altering the inter-hexamer orientation, Bonding Scheme 4 produced biologically relevant growth rates with slower Gag-Gag off rates compared to Bonding Scheme 3. However, though only the orientation of the dimer bond was altered in Bonding Scheme 4, both interfaces showed biologically relevant growth rates at equivalently decreased off rates, suggesting that the same Gag assembly trends hold between Bonding Schemes 3 and 4.

Bonding Scheme 3 predicts that to achieve punctum growth rates comparable to experiment, the strength of the bond linking hexamers together (dimer-bond) should be stronger than the bond promoting hexamer formation (side-bond). Prior to puncta formation, a stronger dimer-bond would yield a sea of Gag dimers which would then need to coalesce into hexamers, whereas a stronger side-bond would produce many independent hexamers that would need to link together to form a hexameric lattice. The building block of the CA capsid in the mature HIV virion has been thought to be a trimer-of-dimers [25,40], but the early stage lattice structure of the immature virion is less clear. It was proposed that MA can form ordered structures during virion assembly [17] but this has not been shown for full length Gag. The model presented here suggests that a trimer-of-dimers is also the basic building block for the immature Gag lattice. This is indicated by the regimes which produced puncta growth rates in agreement with experiment (Fig 5) combined with the results of the monomer-dimer distribution (Fig 6). The model produced puncta growth rates comparable to experiment for a variety of different off rates for the dimer and side-bonds, but for a majority of those off rates the ratio of dimers to monomers was heavily skewed towards monomers. Experimental work using Gag-Gag crosslinking showed that there is a proportion of both dimeric and monomeric Gags in the cytoplasm, with the amount of dimers and higher order multimers forming at the plasma membrane [3]. Thus, the computational results with a stronger dimer-bond compared to the side-bond most accurately match the available data.

A second result of the simulations is the impact of seeding puncta formation by the genomic RNA which potentially brings Gag to the membrane. The model predicts that Gag-Gag off rates which produce feasible puncta growth rates necessitate a nucleus of greater than 50 Gags coming together to initiate a stable punctum that will continue to grow (Fig 7). Though an aggregate of this size is possible, it is unlikely. The simulations which mimicked RNA acting as a seed for formation of a Gag assembly showed formation of a stable punctum with as few as 14 Gags. This is consistent with experimental evidence of Gag interacting with RNA in the cytosol [4] and data showing that RNA appears at the plasma membrane minutes before a Gag punctum is detected [5]. It is thus possible that RNA acts to concentrate Gags at a site on the plasma membrane to induce formation of an immature virus particle.

Finally, both the computational simulations and the analytical model indicate that the observation of Gag puncta having a fairly uniform size distribution and containing a similar number of Gag molecules, is not due simply to reaching a kinetic steady-state in which the number of Gags being added to a growing punctum balances those which dissociate. The simulations showed that for a given off rate and a fixed background concentration of Gags a kinetic steady-state was possible, but with the addition of more Gags into the system the punctum transitions from a steady regime to a growth regime. This is also indicated in the analytical model which shows the growth rate of Gags to be unbounded in the 2D case. Because of this result, all of our simulations require that the curvature of the plasma membrane must occur during Gag assembly and that this is the factor that limits the size of a virion and the number of Gags it contains. Thus, we predict that membrane curvature must be contemporaneous and not after Gag recruitment. The simulations approximating membrane curvature showed the typical sigmoidal shape of puncta assembly, ending in a plateau near 5000 Gags. The same result was found in the analytical model. A comparison of the simulation results and the analytical model showed good agreement, suggesting a role for curvature in immature virion formation. Nevertheless, due to the simplicity of our model and the method by which curvature is introduced, it is possible that there are other factors which act in concert with or in place of membrane curvature to produce uniform viral particles. Recent studies have implicated the host factor angiomotin in promoting viral assembly by aiding membrane curvature, showing that cells lacking angiomotin fail to form spherical particles [41]. Another possibility is that the ESCRT machinery is recruited to an assembling virion at a well-defined state, scissioning off the viral particle well prior to forming a complete assembly of 5000 Gags. Carlson et al. [12,14] measured the completeness of the Gag shell in released immature virions finding that Gag covered 70% of the virion surface corresponding to approximately 2400 ± 700 Gag molecules. When virion budding was arrested by using Gag containing a mutation in the PTAP motif which does not recruit the ESCRT machinery, the virion budding sites showed a more complete Gag lattice shell corresponding to 3800 ± 770 Gags per virion. The simulations presented in this work do not explicitly include the ESCRT machinery and therefore we allow for the possibility for scission by ESCRTs as a primary factor in producing uniform viral particles.

The model presented here was inspired by known details of Gag-Gag interactions while at the same time coarse enough to allow for simulation on realistic timescales. We investigated generalized Gag-Gag bonding schemes and corresponding parameter spaces with the aim of reproducing experimental growth rates with correct lattice structure. Even at this level of coarseness, simulation results recapitulated kinetics and dynamics of puncta formation and allowed for insight into questions surrounding this process, such as RNA seeding and the role of membrane curvature. Future work will aim to refine the Gag-Gag bonding scheme as more becomes known about interactions between different domains of the full-length Gag molecule. A more sophisticated membrane model would be necessary to examine the role of lipid rafts involved in Gag assembly [42], the mechanism by which Gag induces membrane curvature [43], and the influence that membrane curvature has on assembly dynamics [44].

## Supporting information

S1 Fig Spherical cap schematic for the analytical model. R is the radius of the sphere, h is the height of the cap, and a is the radius of the base. b = R–h.
(DOCX)

S2 Fig Orientational distribution for bonded Gags in Bonding Scheme 1. The sharp peaks at 60° and 180° are indicative of a hexameric lattice.
(DOCX)

S3 Fig Simulation of a seeded puncta of Gags modeled with 1 dimer interface and two identical side interfaces. The puncta initially contained 458 Gags on a 1 μm x 1μm membrane patch with 5625 background Gags. After 1 s of simulation time, the initial seeded puncta had nearly dissolved.
(DOCX)

S4 Fig Radial distribution function for Bonded Gags in Bonding Scheme 3. The first peak in the radial distribution function corresponds to individual dimer and side-bonds, which have equilibrium distances at 2.7nm. The second peak corresponds to the second bonding shell around Gags that are present in the punctum.
(DOCX)

S5 Fig Growth rate curves for 3 trials of Bonding Scheme 3 starting from seeded puncta of 2500 Gags with 5000 background Gags. Side-bonding off rate = 125 Gags/s, dimer-bond off-rate = 10^4^ Gags/s. The average growth rate for the 3 trials is 25.1 Gags/s with a standard deviation of 8.2.
(DOCX)

S6 Fig Growth rate contour in Bonding Scheme 3 as a function of side-bond and dimer-bond off rates. Simulations were run for 10 s on a 2.0 μm x 2.0 μm membrane patch with a seeded puncta of 1250 Gags with 5000 background Gags. Growth rates were measured by a linear fit of the number of Gags in the puncta vs. time. Squares represent the simulations performed. Each data point is the average of 3 independent simulations.
(DOCX)

S7 Fig Background concentration at steady-state. Simulations start from an initial seeded punctum of 5000 Gags with varying background concentrations. Side-bond off rate = 5000 Gags/s, dimer-bond off rate = 500 Gags/s.
(DOCX)

S8 Fig Growth rates from the simulations shown in S7 Fig. A linear fit is used to estimate a background concentration of 4479 Gags would result in a zero growth rate.
(DOCX)

S9 Fig Unbounded growth in 2D. Simulations start from an initial seeded punctum of 88 Gags with a background concentration of 5000 Gags. Side-bond off rate = 500 Gags/s, dimer-bond off rate = 5000 Gags/s.
(DOCX)
