# Supplementary figures and images for: Modeling the dynamics and kinetics of HIV-1 Gag during viral assembly
Source: PLoS One. 2018 Apr 20;13(4):e0196133. doi: 10.1371/journal.pone.0196133 (PMC5909904; doi:10.1371/journal.pone.0196133)

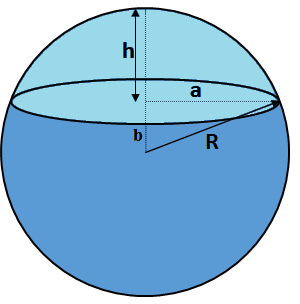

Supplement: S1 Fig — R is the radius of the sphere, h is the height of the cap, and a is the radius of the base. b = R–h. (DOCX) [file pone.0196133.s001.docx]

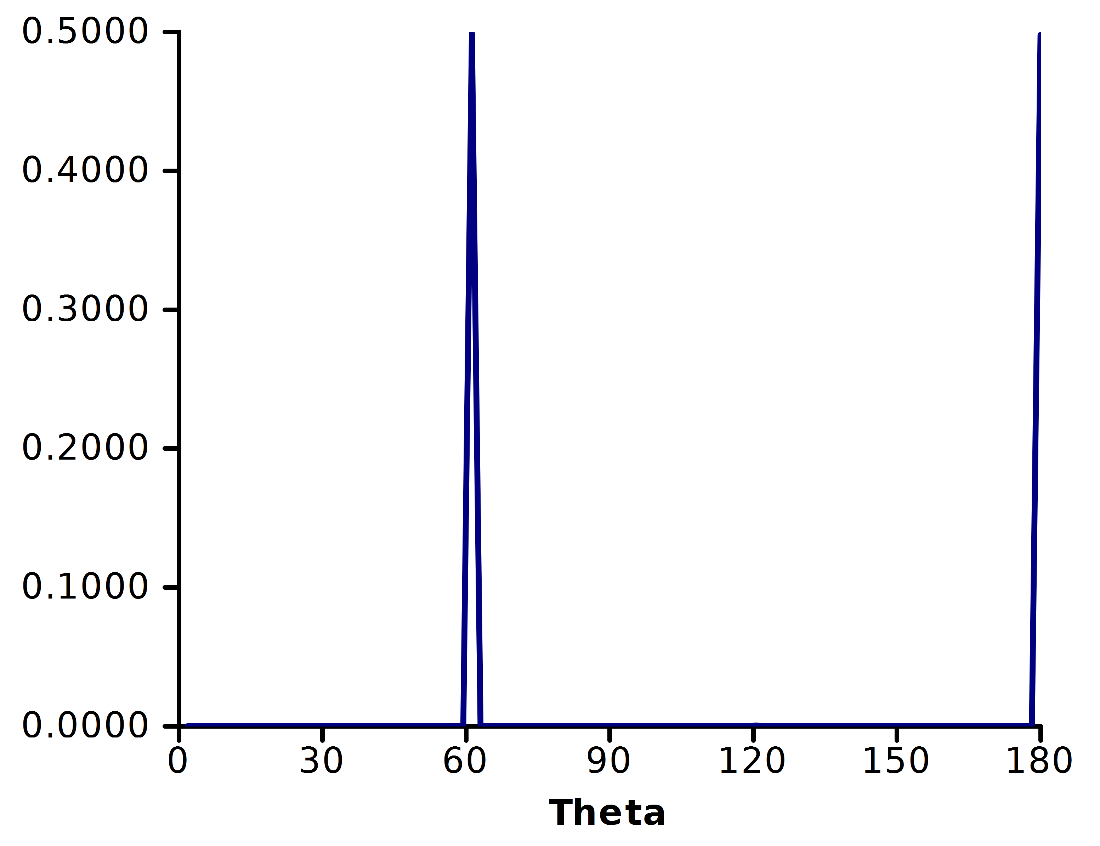

Supplement: S2 Fig — The sharp peaks at 60° and 180° are indicative of a hexameric lattice. (DOCX) [file pone.0196133.s002.docx]

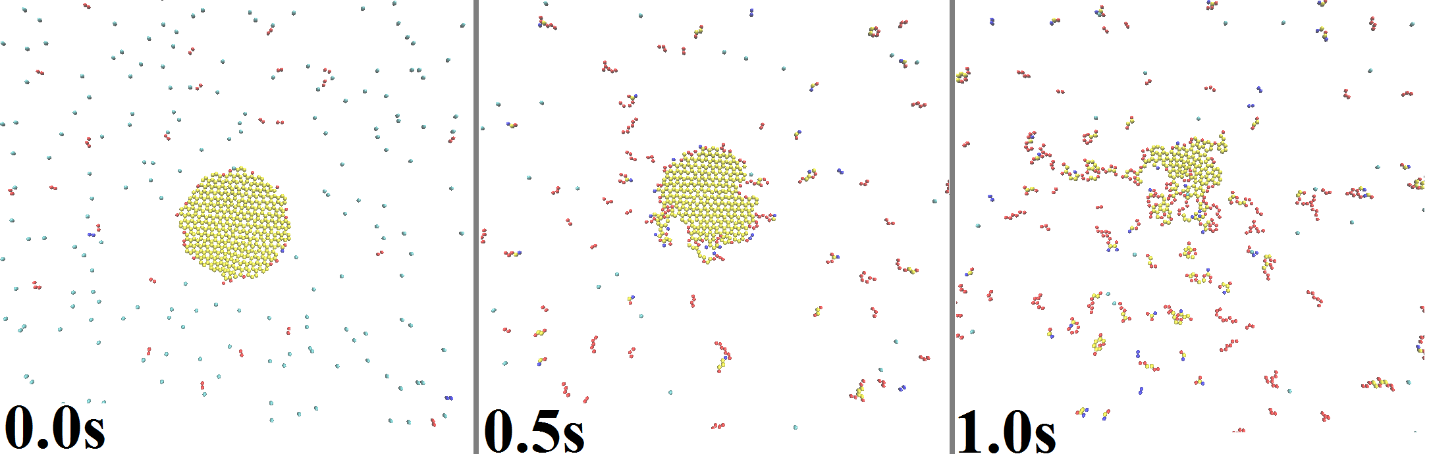

Supplement: S3 Fig — The puncta initially contained 458 Gags on a 1 μm x 1μm membrane patch with 5625 background Gags. After 1 s of simulation time, the initial seeded puncta had nearly dissolved. (DOCX) [file pone.0196133.s003.docx]

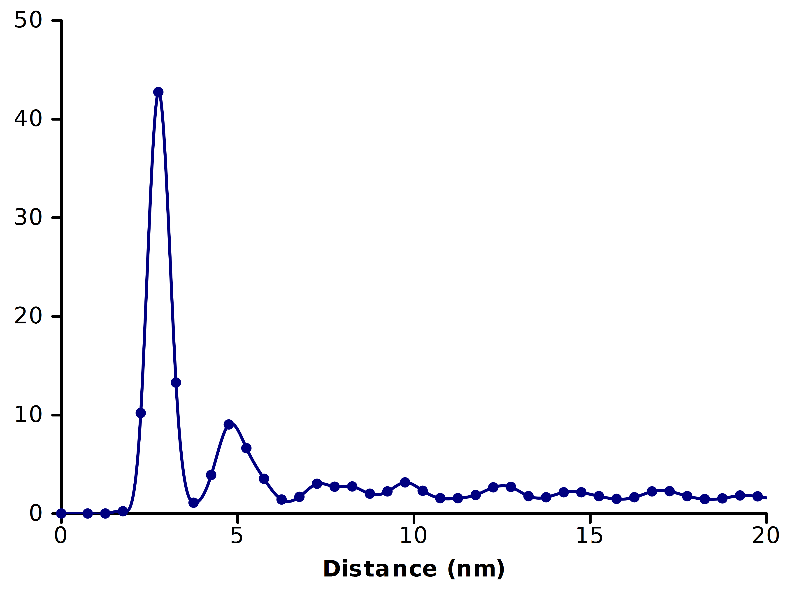

Supplement: S4 Fig — The first peak in the radial distribution function corresponds to individual dimer and side-bonds, which have equilibrium distances at 2.7nm. The second peak corresponds to the second bonding shell around Gags that are present in the punctum. (DOCX) [file pone.0196133.s004.docx]

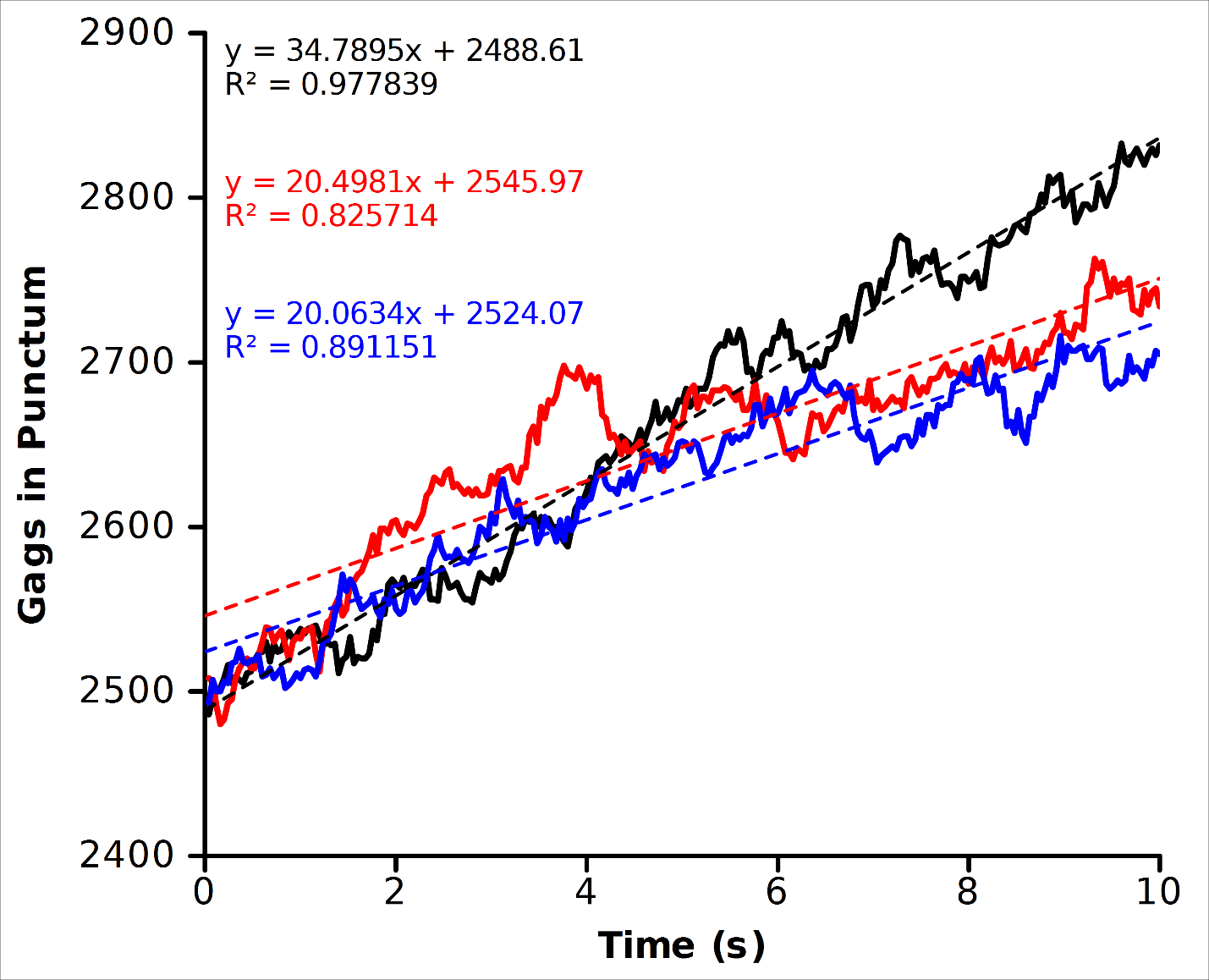

Supplement: S5 Fig — Side-bonding off rate = 125 Gags/s, dimer-bond off-rate = 104 Gags/s. The average growth rate for the 3 trials is 25.1 Gags/s with a standard deviation of 8.2. (DOCX) [file pone.0196133.s005.docx]

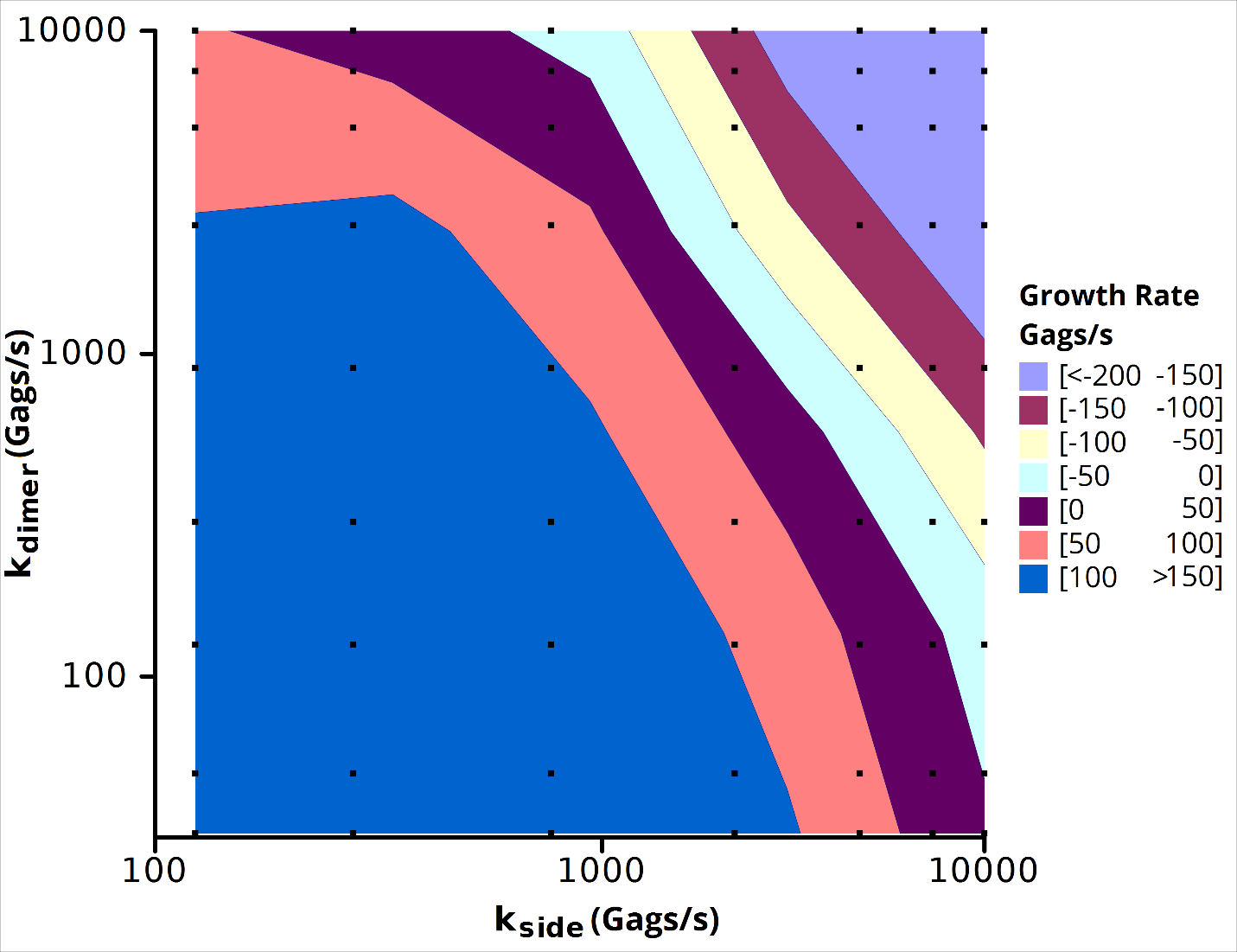

Supplement: S6 Fig — Simulations were run for 10 s on a 2.0 μm x 2.0 μm membrane patch with a seeded puncta of 1250 Gags with 5000 background Gags. Growth rates were measured by a linear fit of the number of Gags in the puncta vs. time. Squares represent the simulations performed. Each data point is the average of 3 independent simulations. (DOCX) [file pone.0196133.s006.docx]

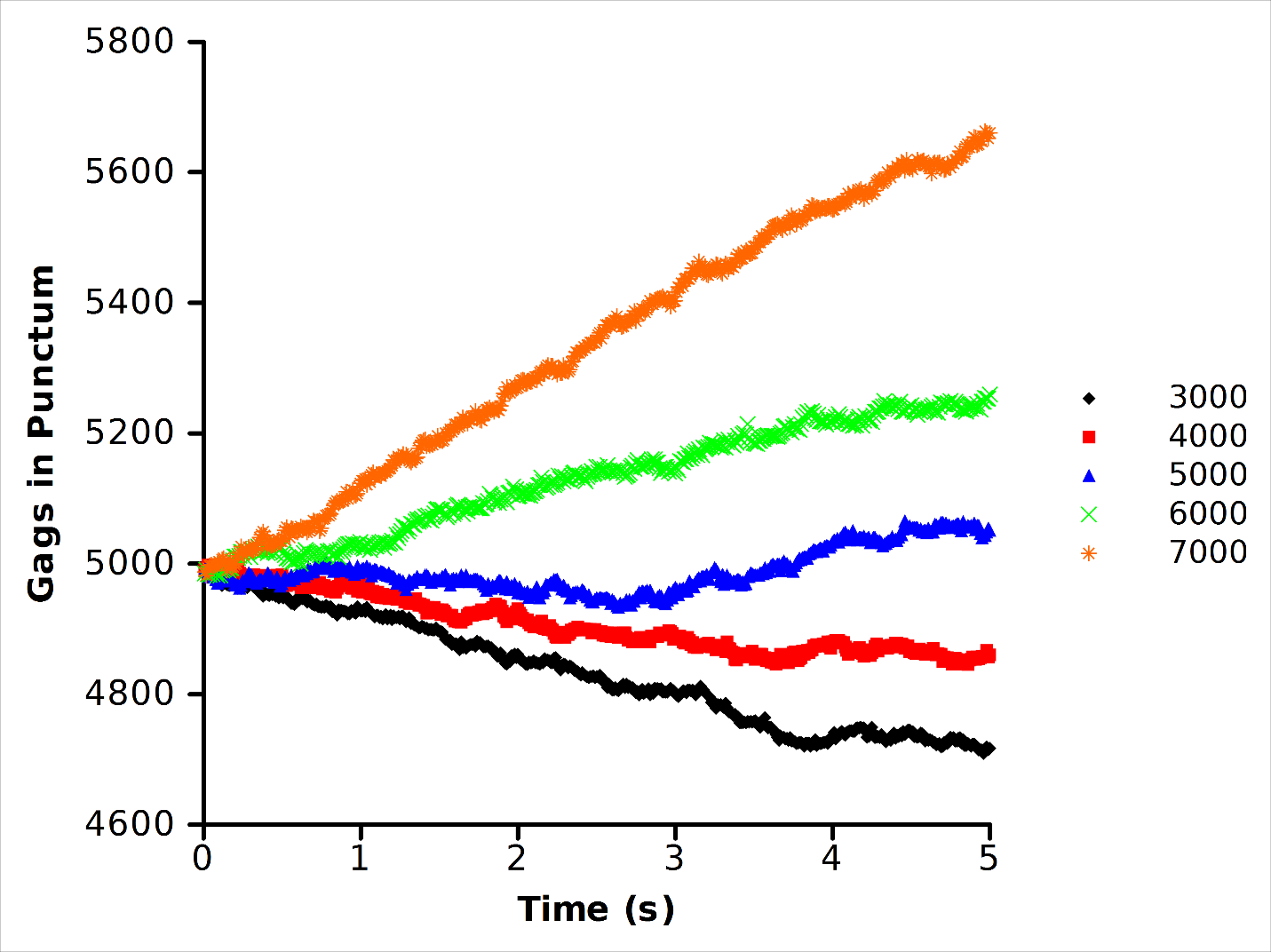

Supplement: S7 Fig — Simulations start from an initial seeded punctum of 5000 Gags with varying background concentrations. Side-bond off rate = 5000 Gags/s, dimer-bond off rate = 500 Gags/s. (DOCX) [file pone.0196133.s007.docx]

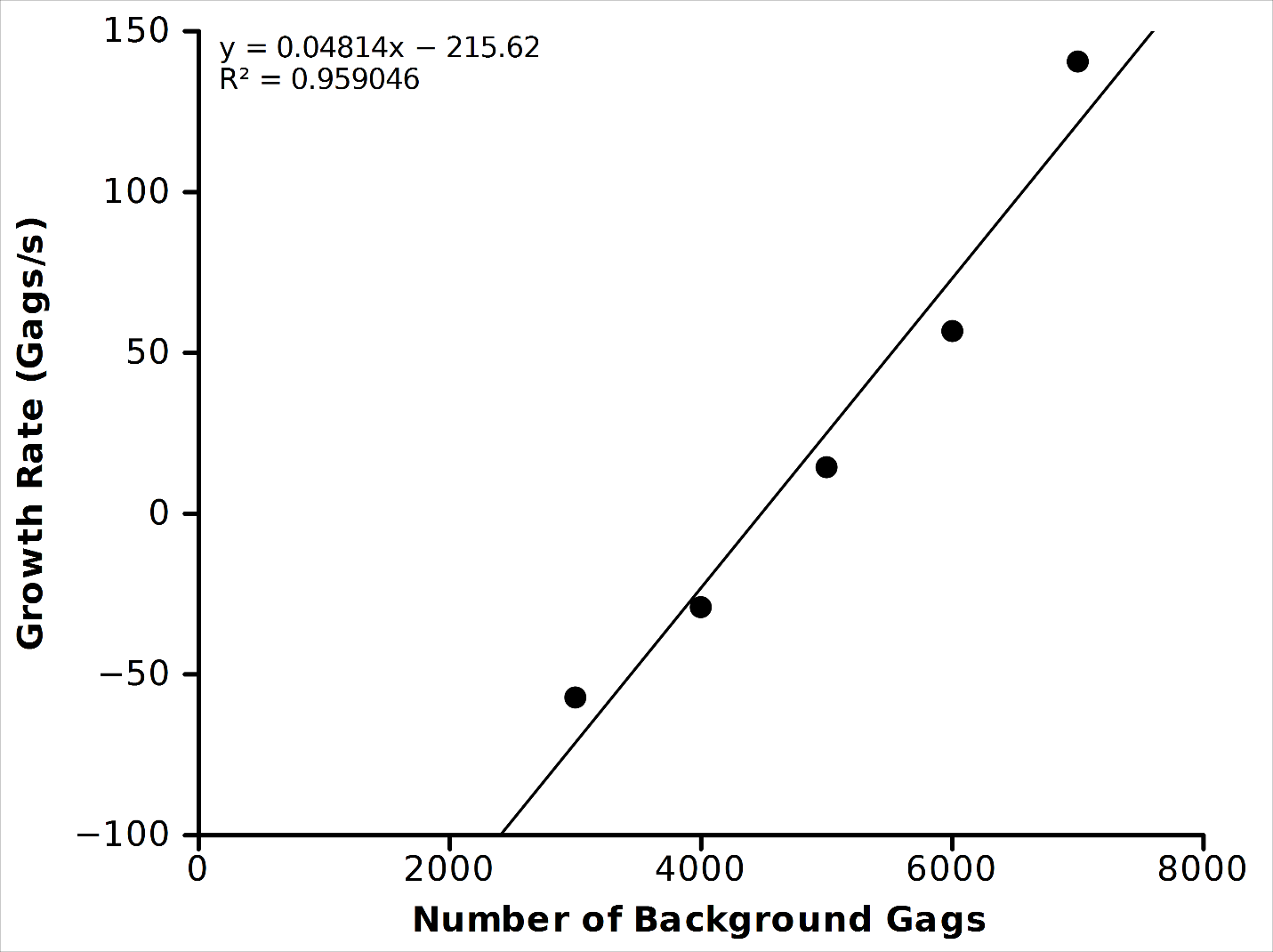

Supplement: S8 Fig — A linear fit is used to estimate a background concentration of 4479 Gags would result in a zero growth rate. (DOCX) [file pone.0196133.s008.docx]

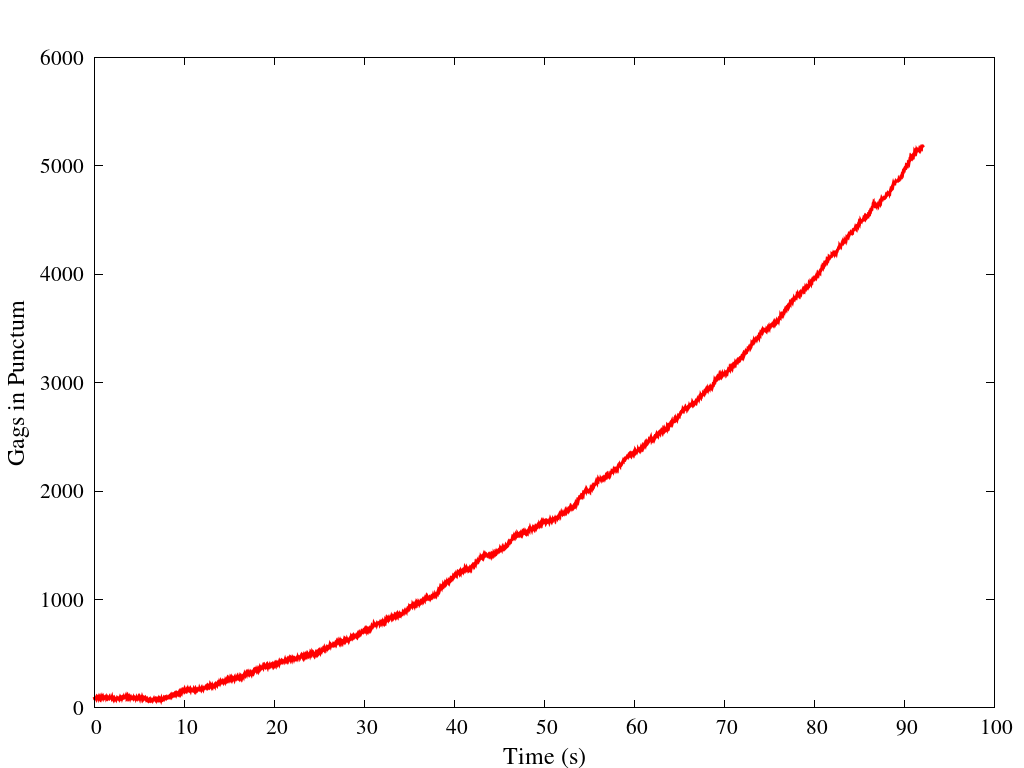

Supplement: S9 Fig — Simulations start from an initial seeded punctum of 88 Gags with a background concentration of 5000 Gags. Side-bond off rate = 500 Gags/s, dimer-bond off rate = 5000 Gags/s. (DOCX) [file pone.0196133.s009.docx]
